# A hypoxia-responsive calixarene supramolecular system enables redox-regulated matrix remodeling for postmenopausal osteoporosis

**DOI:** 10.1016/j.mtbio.2026.103685

**Published:** 2026-09-23

**Authors:** Gan Luo, Houzhi Yang, Ze-Han Wang, Jianlin Cui, Qiong Tang, Yonggang Fan, Hong Yang, Yubin Long, Shanshan Li, Dong-Sheng Guo, Tianwei Sun, Xin Jin

**Affiliations:** a School of Medicine, Nankai University, Tianjin, China; b College of Chemistry, State Key Laboratory of Elemento-Organic Chemistry, Key Laboratory of Functional Polymer Materials (Ministry of Education), Frontiers Science Center for New Organic Matter, Collaborative Innovation Center of Chemical Science and Engineering, Nankai University, Tianjin, 300071, China; c Department of Spinal Surgery, Tianjin Union Medical Center, The First Affiliated Hospital of Nankai University, Tianjin, 300121, China; d Tianjin Institute of Spinal Surgery, Tianjin, 300121, China; e Department of Respiratory, Tianjin Union Medical Center, The First Affiliated Hospital of Nankai University, Tianjin, 300121, China; f Department of Orthopedics, Chengdu Integrated Traditional Chinese Medicine &Western Medicine Hospital, Chengdu First People's Hospital, Chengdu, 610016, China; g Academy for Advanced Interdisciplinary Studies, Nankai University, Tianjin, 300071, China; h Tianjin Medical University, Tianjin, China; i School of Medical and Life Sciences, Chengdu University of Traditional Chinese Medicine, Chengdu, 611137, China; j Department of Spinal Surgery, The Central Hospital of Shaoyang, Shaoyang, Hunan, 42200, China

**Keywords:** Supramolecular system, Calixarene, Hypoxia-responsive regulation, Bone microenvironment, Matrix remodeling

## Abstract

Postmenopausal osteoporosis (PMOP) is characterized by progressive bone loss driven by excessive osteoclast activity, impaired osteogenesis, and maladaptive remodeling of the bone microenvironment. Although raloxifene (RLX) is clinically approved for PMOP, its therapeutic potential is constrained by limited bone selectivity and systemic adverse effects. Here, we report a hypoxia-responsive, bone-targeted supramolecular system (RLX@PnAC4A) that integrates site-selective drug delivery with redox regulation to enable coordinated matrix remodeling within the osteoporotic niche. PnAC4A was engineered through azobenzene functionalization and phosphonate modification, endowing the calixarene host with hypoxia-triggered reducibility, high-affinity bone targeting, and efficient RLX encapsulation. The resulting supramolecular assemblies exhibited stable host–guest interactions and selective drug release under hypoxic conditions. In vitro, RLX@PnAC4A preferentially restored intracellular redox homeostasis under hypoxic and inflammatory stress, enhanced osteogenic differentiation, and suppressed osteoclastogenesis in a microenvironment-dependent manner. Following systemic administration in ovariectomized (OVX) mice, RLX@PnAC4A rapidly accumulated in bone tissue and significantly improved trabecular microarchitecture, bone formation dynamics, and structural integrity compared with free RLX. Transcriptomic profiling further revealed that OVX induced a broadly immune-activated disease program, whereas PnAC4A alone established an extracellular matrix–associated conditioning state. Importantly, RLX@PnAC4A activated a dominant, therapy-specific transcriptional module enriched in extracellular matrix remodeling and pro-regenerative signaling, accompanied by directional rescue of key disease-associated nodes. Collectively, these findings demonstrate that RLX@PnAC4A functions as an active supramolecular regulator, exerting therapeutic efficacy through a hierarchically organized transcriptional architecture that couples bone targeting, redox modulation, and osteoblast–osteoclast coordination. This hypoxia -responsive calixarene platform establishes a mechanistically grounded strategy for precision intervention in postmenopausal osteoporosis.

## Graphical abstract

**Figure:**
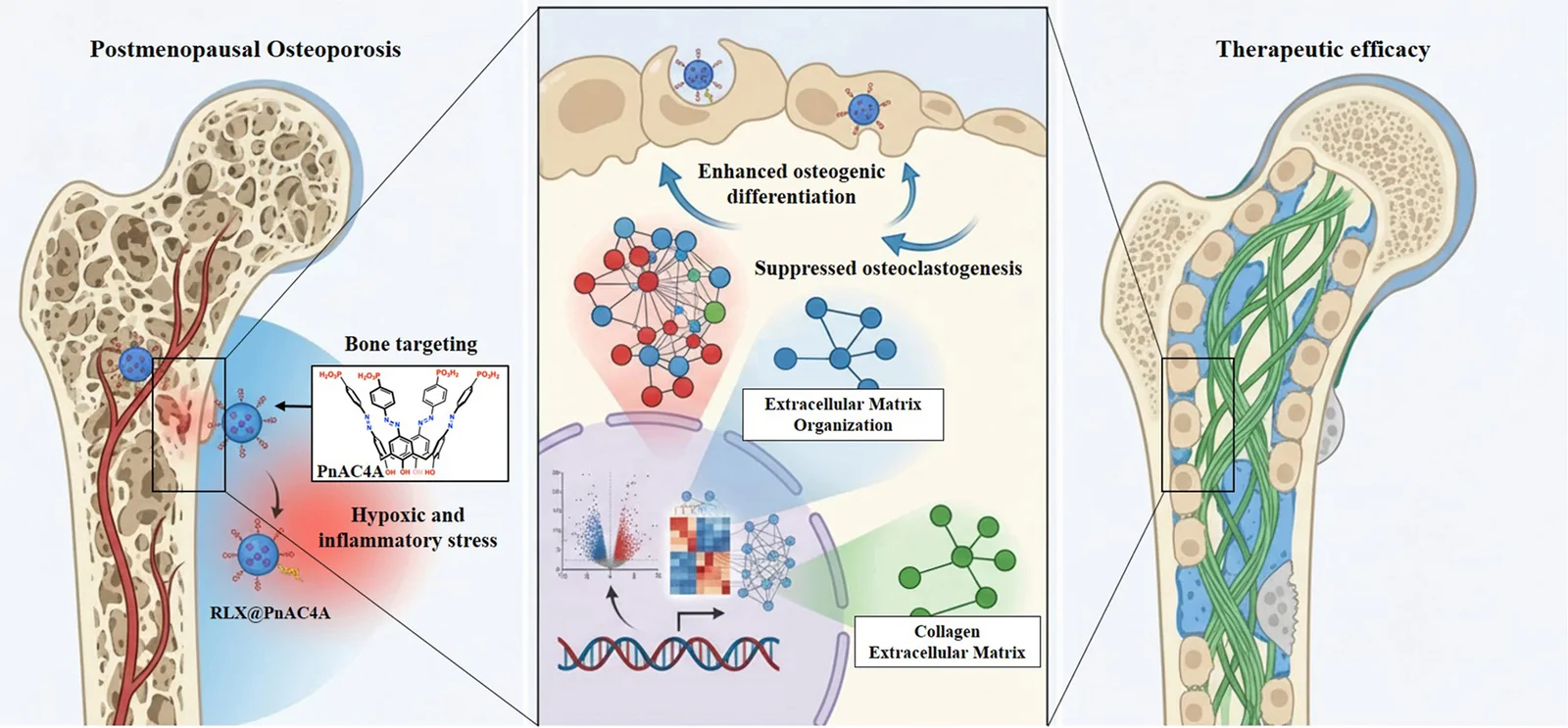

## 1 Introduction

Osteoporosis is a chronic skeletal disorder marked by progressive loss of bone mass and deterioration of microarchitecture, affecting hundreds of millions of postmenopausal women worldwide and imposing an escalating socioeconomic burden in aging societies [1,2]. Postmenopausal osteoporosis (PMOP), primarily driven by estrogen deficiency, is increasingly recognized not merely as a disorder of bone quantity but as a disease of microenvironmental dysregulation [[3], [4], [5]]. Excessive osteoclast activation, elevated oxidative stress, chronic inflammation, and impaired osteogenesis converge to disrupt the tightly coupled process of bone remodeling [6,7]. Although anti-resorptive and anabolic agents, including bisphosphonates, denosumab, teriparatide, and selective estrogen receptor modulators (SERMs), are widely used in clinical practice, long-term therapeutic outcomes remain unsatisfactory owing to limited tissue specificity, systemic adverse effects, and poor patient adherence [[8], [9], [10], [11], [12]]. These limitations highlight a critical unmet need for therapeutic strategies capable of selectively targeting diseased bone while actively correcting the pathological bone microenvironment.

Raloxifene (RLX), a clinically approved SERM, exerts estrogen receptor–dependent protective effects on bone and reduces vertebral fracture risk in postmenopausal women [13,14]. However, its clinical utility is substantially constrained by extremely low oral bioavailability, extensive first-pass metabolism, and off-target estrogenic effects in non-skeletal tissues [15]. More fundamentally, RLX monotherapy primarily modulates osteoblastic signaling and does not directly address the hypoxic, oxidative, and inflammatory stresses that define the osteoporotic bone niche and critically influence osteoblast–osteoclast coupling [6,16]. These considerations suggest that enhancing RLX efficacy requires not only improved bone-selective delivery, but also context-aware modulation of the diseased microenvironment in which bone remodeling occurs. Recent biomaterial strategies have begun to address bone-microenvironment dysfunction through coordinated regulation of osteoimmunity, angiogenesis, and osteogenesis. A pH-responsive mussel-inspired adhesive improved osteoporotic bone–implant integration through coupled immune, vascular, and osteogenic regulation [17]. Similarly, an integrin α2β1-targeting biomimetic coating enhanced interfacial osseointegration by promoting M2 macrophage polarization and vascularized bone regeneration [18]. Nevertheless, these locally applied, implant-associated platforms do not address systemic bone-selective delivery or oxygen-responsive drug deployment in non-implant-associated PMOP.

Recent advances in supramolecular chemistry have provided powerful design principles for constructing intelligent therapeutic systems with programmable responsiveness and modular functionality [[19], [20], [21]]. Among macrocyclic hosts, calixarenes often regarded as third-generation supramolecular scaffolds following cyclodextrins and cucurbiturils, offer a rigid cup-shaped architecture for stable host–guest complexation combined with exceptional chemical tunability [[22], [23], [24]]. In principle, functional calixarene platforms can be engineered to integrate multiple therapeutic requirements, including targeted accumulation, stimulus-responsive release, and microenvironmental adaptability. Notably, azobenzene motifs confer hypoxia-sensitive reducibility, while phosphonate groups exhibit strong affinity for hydroxyapatite-rich bone surfaces [25,26]. Despite these attractive features, whether multifunctional calixarene-based supramolecular systems can actively reprogram the osteoporotic bone microenvironment and restore balanced remodeling in vivo remains largely unexplored.

Here, we report a hypoxia-responsive, bone-targeted supramolecular system (RLX@PnAC4A) in which RLX is encapsulated within a phosphonate-modified azobenzene calixarene host. This system was designed to integrate stable drug complexation, hypoxia-triggered release, and preferential accumulation within osteoporotic bone. We systematically investigated its supramolecular properties, redox-regulatory capacity, and functional effects on osteoblast and osteoclast activities under hypoxic and inflammatory conditions, as well as its therapeutic efficacy in ovariectomized (OVX) mice. By combining in vivo phenotypic analyses with transcriptomic profiling, this study elucidates how bone-targeted supramolecular conditioning and payload delivery cooperatively reshape the osteoimmune microenvironment, thereby establishing a mechanistically grounded framework for precision intervention in postmenopausal osteoporosis.

## 2 Results

### 2.1 RLX@PnAC4A supramolecular properties and cellular redox regulation

PnAC4A was engineered as a hypoxia-responsive, bone-affinitive calixarene host by integrating reducible azobenzene linkers with phosphonate functionalities, thereby enabling both stable cargo encapsulation and stimulus-triggered cargo release (Fig. 1A). In aqueous solution, PnAC4A formed quasi-spherical nanoassemblies, as qualitatively visualized by transmission electron microscopy (Fig. 1A). Intensity-weighted DLS analysis showed unimodal size distributions for both PnAC4A and RLX@PnAC4A (Fig. 1B). The colloidal stability of the final RLX@PnAC4A formulation was further evaluated under serum-containing conditions. Its hydrodynamic diameter remained approximately 150–160 nm, while the PDI remained approximately 0.28–0.32 over the 36-h observation period, without a marked time-dependent increase in either parameter (Fig. 1C). These findings support the short-term colloidal stability of RLX@PnAC4A under the tested serum-containing conditions. Zeta-potential measurements further characterized the surface charge of the PnAC4A and RLX@PnAC4A assemblies (Fig. 1D).

**Fig. 1:**
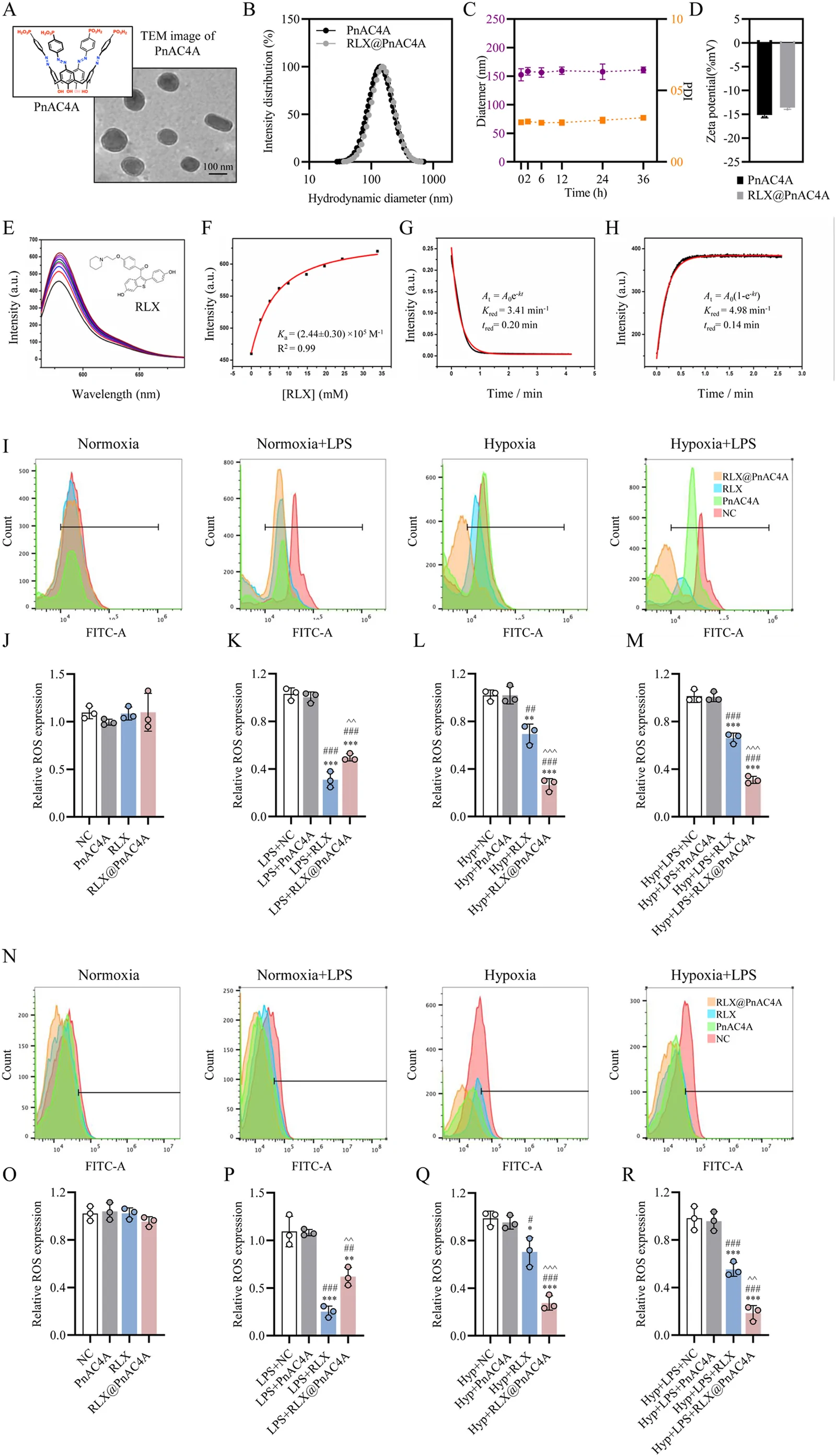
**Supramolecular characterization of RLX@PnAC4A and hypoxia/inflammation-dependent regulation of intracellular ROS**. (A) Chemical structure of the hypoxia-responsive phosphonate azocalixarene host PnAC4A and representative transmission electron microscopy image showing the morphology of PnAC4A nanoassemblies. (B) Intensity-weighted DLS size distributions of PnAC4A and RLX@PnAC4A (C) Time-dependent hydrodynamic diameter and polydispersity index of RLX@PnAC4A during 36 h of incubation under serum-containing conditions. (D) The zeta potential of PnAC4A and RLX@PnAC4A. (E) Competitive fluorescence titration of RLX in the presence of RhB (1.00 μM) and PnAC4A (3.00 μM) in HEPES; (F) The associated titration curve at λem = 575 nm was fitted according to the 1:1 competitive binding model. (G) The reduction kinetics (the black line) of PnAC4A (10.0 μM) monitored by absorbance at 420 nm as a function of time under SDT-induced reducing conditions (addition of 10.00 mM SDT) and the corresponding fitting curve (the red line) according to a quasi-first order reaction decay model; (H) The release kinetics (the black line) of RhB@PnAC4A (10.00/20.0 μM) monitored by fluorescence intensity at 575 nm as a function of time under SDT-induced reducing conditions and the corresponding fitting curve (the red line) according to a quasi-first order reaction model. All experiments were performed in HEPES buffer (10 mM, pH 7.4) at 37 °C under SDT-induced reducing conditions. (I) Representative flow cytometry histograms of intracellular ROS in MC3T3-E1 cells under normoxia, normoxia + LPS, hypoxia, and hypoxia + LPS. (J-M) Quantification of relative ROS levels in MC3T3-E1 cells under normoxia (J), normoxia + LPS (K), hypoxia (L), and hypoxia + LPS (M). (N) Representative flow cytometry histograms of intracellular ROS in primary BMMs under the same conditions and treatments as in (I). (O–R) Quantification of relative ROS levels in primary BMMs under normoxia (O), normoxia + LPS (P), hypoxia (Q), and hypoxia + LPS (R). Data are presented as mean ± S.D. from n = 3 independent experiments. Statistical significance was assessed by one-way ANOVA followed by Tukey's multiple-comparisons test. *P < 0.05, **P < 0.01, ***P < 0.001 vs. NC group; #P < 0.05, ##P < 0.01, ###P < 0.001 vs. PnAC4A group; ^P < 0.05, ^^P < 0.01, ^^^P < 0.001 vs. RLX group.

We next evaluated the host–guest interaction between PnAC4A and the therapeutic payload raloxifene (RLX) using a fluorescence competitive titration experiment. PnAC4A was pre-complexed with the dye RhB, resulting in fluorescence quenching of the dye. Upon gradual addition of RLX, the fluorescence intensity progressively increased, indicating that the RhB dye initially bound to PnAC4A was displaced due to the binding of RLX to PnAC4A (Fig. 1E). The titration data were well fitted with a 1:1 competitive binding model, yielding an apparent association constant of *K*_a_ = (2.44 ± 0.30) × 10^5^ M^−1^ with a fitted R^2^ = 0.99 (Fig. 1F). These results indicate that PnAC4A provides sufficient molecular recognition to stably accommodate RLX. In addition, we evaluated the hypoxia-responsive behavior of PnAC4A. SDT was used as an in vitro mimic of azoreductase, and the absorbance at 420 nm was monitored by UV–visible spectroscopy. The decrease in absorbance indicated the reduction of the azo bond. To investigate drug release, the dye RhB was employed as a model drug, as its binding constant with PnAC4A is comparable to that of the actual drug (RLX). Upon the addition of SDT, the fluorescence rapidly recovered, indicating that the cargo can be quickly released under reductive conditions (Fig. 1G and H).

We then examined whether PnAC4A and RLX@PnAC4A regulate intracellular oxidative stress in MC3T3-E1 osteoblast-lineage cells and bone marrow–derived macrophages (primary BMMs) using flow cytometry–based ROS profiling. Representative histogram overlays demonstrated minimal separation among groups under normoxia in MC3T3-E1 (Fig. 1I) and primary BMMs cells (Fig. 1N), consistent with the quantification showing comparable ROS levels across NC, PnAC4A, RLX, and RLX@PnAC4A (Fig. 1J and O).

Upon LPS stimulation under normoxia, ROS increased in controls, whereas both RLX and RLX@PnAC4A significantly reduced ROS in MC3T3-E1 cells (Fig. 1K) and primary BMMs (Fig. 1P). Notably, in this normoxic inflammatory setting, free RLX produced a stronger ROS suppression than RLX@PnAC4A, suggesting a limited added benefit of the carrier under normoxic conditions. In contrast, hypoxia alone produced an oxidative shift in controls, and RLX@PnAC4A displayed the most pronounced ROS-lowering activity (Fig. 1L) and primary BMMs (Fig. 1Q). This advantage was also observed under hypoxia + LPS, where RLX@PnAC4A consistently achieved the deepest suppression of ROS in both cell types (Fig. 1M and R). These data demonstrate that PnAC4A forms stable supramolecular nanoassemblies and that RLX@PnAC4A most effectively restores redox homeostasis specifically under hypoxic conditions.

### 2.2 Effects of RLX@PnAC4A on osteogenic and osteoclastic cellular functions

Building on the hypoxia-responsive supramolecular behavior and redox-buffering activity of RLX@PnAC4A, we next evaluated whether this supramolecular formulation functionally rewires osteogenic and osteoclastic programs. We modeled normoxia versus hypoxia, with or without LPS-driven inflammatory challenge, and compared NC, PnAC4A, free RLX, and RLX@PnAC4A across MC3T3-E1 and primary BMMs cells.

Cell-cycle analysis of MC3T3-E1 cells showed no change of cytostatic arrest across treatments under either normoxia or hypoxia, with comparable G1/S/G2–M distributions among groups (Fig. 2A and B). Consistently, apoptosis remained low under normoxia and was not elevated by PnAC4A or RLX@PnAC4A (Fig. 2A and C), supporting cytocompatibility of the supramolecular system. Under hypoxia, apoptosis was significantly reduced by PnAC4A and RLX@PnAC4A (Fig. 2D and F), indicating improved tolerance to oxygen-limited stress. We then assessed osteogenic function by matrix mineralization. Under normoxia, free RLX enhanced mineral deposition, whereas RLX@PnAC4A produced a more moderate increase (Fig. 2G and H). A similar pattern was observed under normoxia + LPS (Fig. 2G and I). Strikingly, under hypoxia, the efficacy was inverted. RLX@PnAC4A elicited the strongest mineralization response (Fig. 2J and K), and this advantage became most pronounced under combined hypoxia + LPS, where RLX@PnAC4A markedly preserved and amplified mineral deposition compared with free RLX and PnAC4A alone (Fig. 2J and L). Thus, RLX@PnAC4A exhibits a microenvironment-dependent functional gain, preferentially manifesting under hypoxic and inflammatory conditions.

**Fig. 2:**
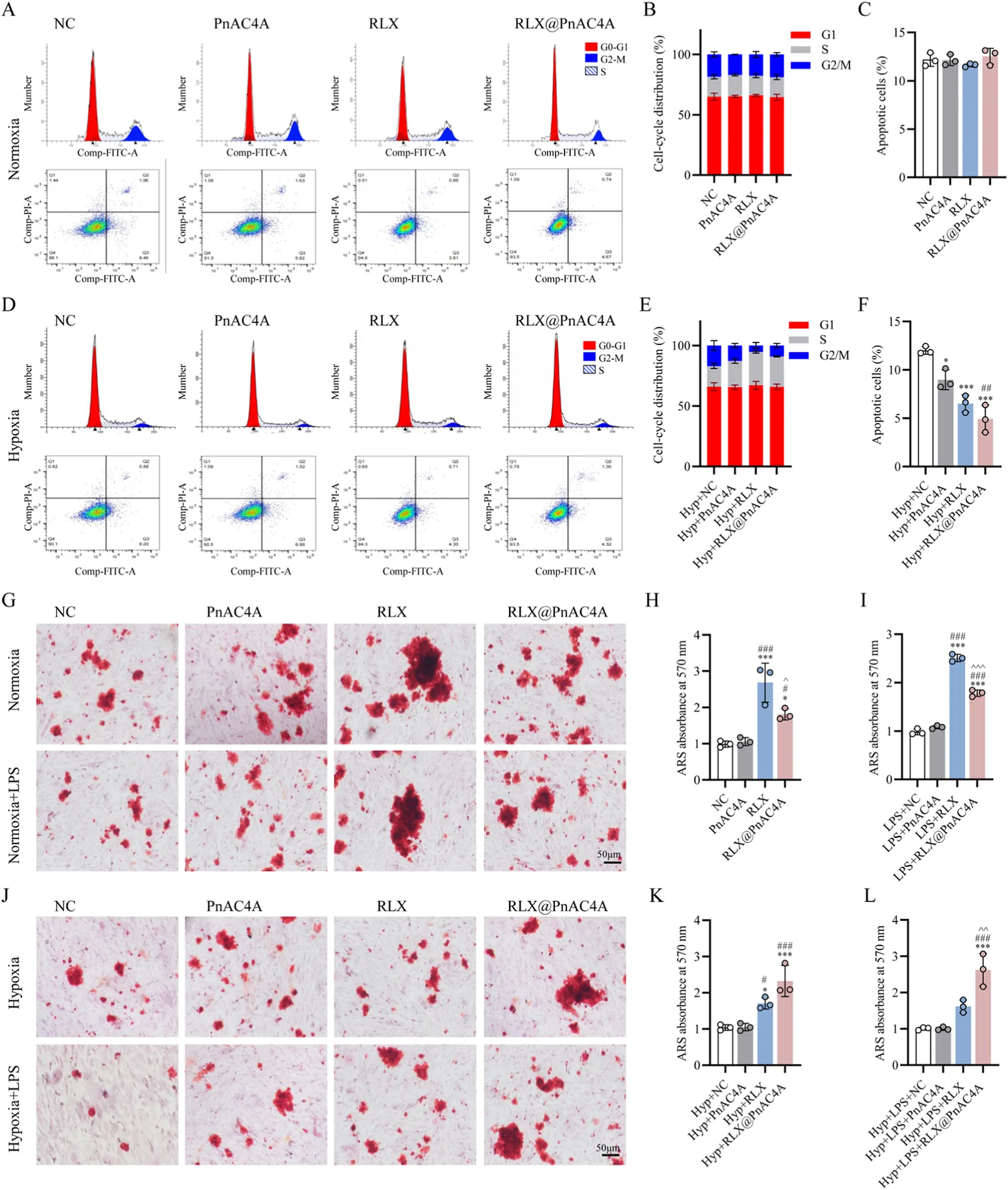
**RLX@PnAC4A preserves cytocompatibility and preferentially promotes osteogenic mineralization under hypoxia and hypoxic inflammation.** (A) Representative flow-cytometry profiles of MC3T3-E1 cells cultured under normoxia and treated with NC, PnAC4A, RLX, or RLX@PnAC4A. Upper panels: DNA-content histograms for cell-cycle analysis; lower panels: Annexin V/PI dot plots for apoptosis assessment. (B) Quantification of cell-cycle phase distribution (G1, S, and G2/M) under normoxia corresponding to (A). (C) Quantification of apoptotic rate under normoxia corresponding to (A). (D) Representative flow-cytometry profiles of MC3T3-E1 cells cultured under hypoxia following the indicated treatments. Upper panels: DNA-content histograms; lower panels: Annexin V/PI dot plots. (E) Quantification of cell-cycle phase distribution under hypoxia corresponding to (D). (F) Quantification of apoptotic rate under hypoxia corresponding to (D). (G) Representative Alizarin Red S (ARS) staining images of mineralized matrix in MC3T3-E1 cultures under normoxia (top row) and normoxia + LPS (bottom row) following treatment with NC, PnAC4A, RLX, or RLX@PnAC4A. (H, I) Colorimetric quantification of ARS staining (absorbance at 570 nm) under normoxia (H) and normoxia + LPS (I) corresponding to (G). (J) Representative ARS staining images under hypoxia (top row) and hypoxia + LPS (bottom row) after the indicated treatments. (K, L) Quantification of ARS staining (absorbance at 570 nm) under hypoxia (K) and hypoxia + LPS (L), corresponding to (J). Data are presented as mean ± S.D. from n = 3 independent experiments. Statistical significance was assessed by one-way ANOVA followed by Tukey's multiple-comparisons test. *P < 0.05, **P < 0.01, ***P < 0.001 vs. NC group; #P < 0.05, ##P < 0.01, ###P < 0.001 vs. PnAC4A group; ^P < 0.05, ^^P < 0.01, ^^^P < 0.001 vs. RLX group.

To further substantiate the functional mineralization phenotypes observed above, early osteogenic differentiation and lineage-specific marker expression were evaluated (Fig. 3). Alkaline phosphatase (ALP) staining revealed that under normoxia and normoxia plus LPS, both free RLX and RLX@PnAC4A increased ALP activity relative to the corresponding controls, with free RLX producing a modestly greater response than RLX@PnAC4A (Fig. 3A–C, and D). In contrast, under hypoxia and hypoxia plus LPS, RLX@PnAC4A elicited the most pronounced increase in ALP activity (Fig. 3B–E, and F). Consistent with these functional readouts, representative immunoblots showed that RLX@PnAC4A increased the osteogenic transcriptional and matrix-associated proteins RUNX2, OSX, and OPN (Fig. 3G and H). Densitometric analyses under normoxia and normoxia + LPS are shown in Fig. 3I–N, whereas the corresponding analyses under hypoxia and hypoxia + LPS are shown in Fig. 3O–T. The effects of RLX@PnAC4A were most pronounced under hypoxic and combined hypoxic/inflammatory condition. To determine whether the osteogenic effects observed in MC3T3-E1 cells were retained in primary osteoprogenitor cells, we further evaluated osteogenic differentiation in primary mouse BMSCs. Early osteogenic differentiation was assessed by ALP staining, whereas late-stage matrix mineralization was evaluated by ARS staining. Under normoxic conditions, free RLX produced a strong increase in ALP activity and mineral deposition, while RLX@PnAC4A elicited a comparatively moderate response. In contrast, under LPS-induced inflammatory stress, hypoxia, and combined hypoxia plus LPS, RLX@PnAC4A markedly enhanced both ALP activity and mineralized matrix formation relative to NC and PnAC4A treatment (Supplementary Figs. S4 and S5). The osteogenic advantage of RLX@PnAC4A was most evident under combined hypoxic and inflammatory stress, where its effects were comparable to or greater than those of free RLX. These findings reproduce the microenvironment-dependent osteogenic response observed in MC3T3-E1 cells and demonstrate that the effect extends to primary mouse BMSCs.

**Fig. 3:**
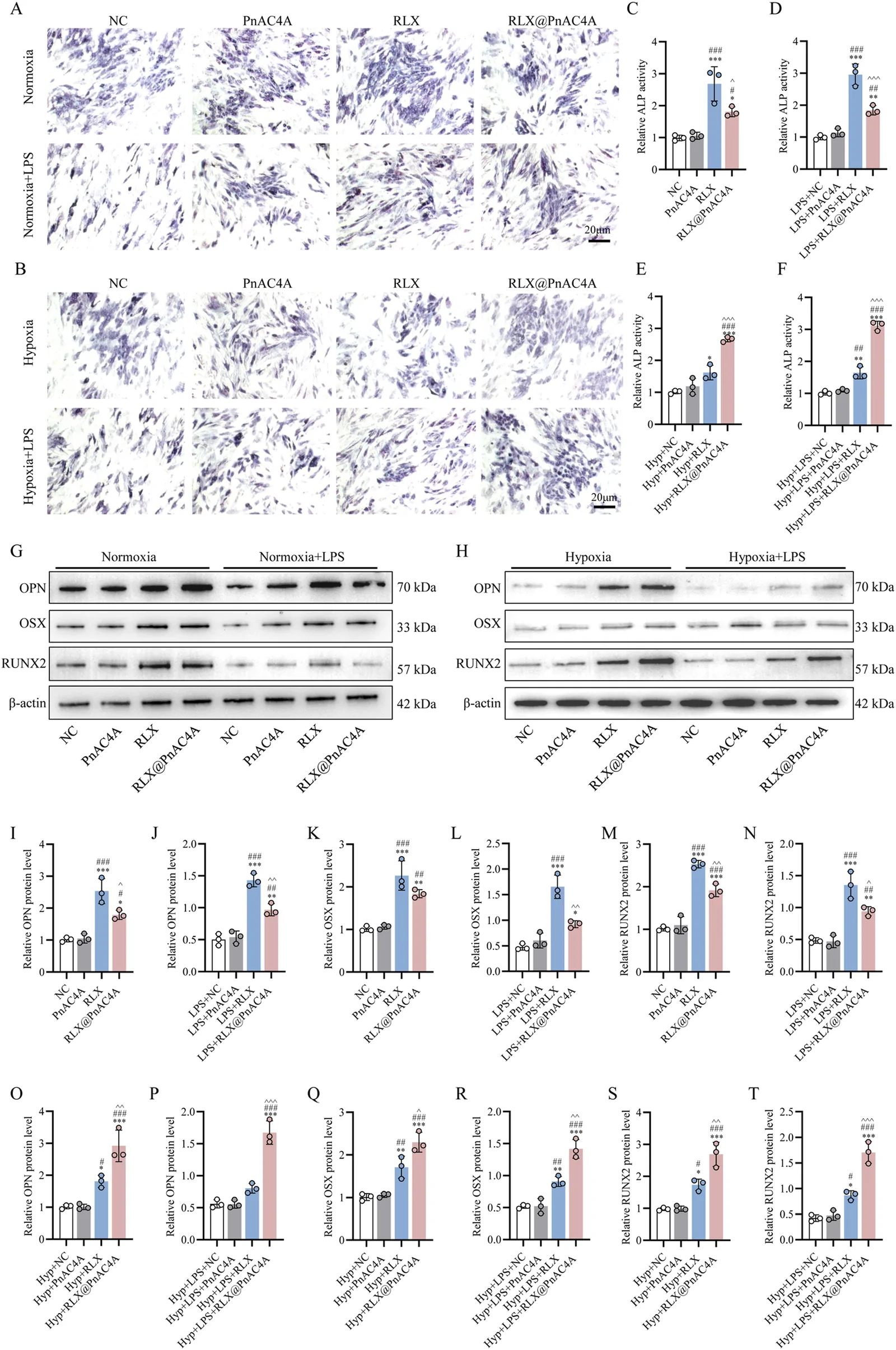
**RLX@PnAC4A sustains osteogenic differentiation and lineage-specific gene expression under hypoxic and inflammatory stress in MC3T3-E1 cells.** (A–B) Representative images of alkaline phosphatase (ALP) staining in MC3T3-E1 cells cultured under normoxic (A) or hypoxic (B) conditions, with or without LPS stimulation, following treatment with NC, PnAC4A, free RLX, or RLX@PnAC4A. (C–F) Quantitative analysis of ALP activity corresponding to panels (A–B), showing relative ALP expression under normoxia (C), normoxia + LPS (D), hypoxia (E), and hypoxia + LPS (F). (G–H) Western blot analysis of osteogenic marker proteins OPN, OSX, and RUNX2 in MC3T3-E1 cells under normoxic (G) and hypoxic (H) conditions, with or without LPS stimulation, following the indicated treatments. β-Actin was used as a loading control. (I–N) Densitometric quantification of OPN, OSX, and RUNX2 protein expression corresponding to panels (G), normalized to β-actin and presented as relative expression levels. (O–T) Densitometric quantification of OPN, OSX, and RUNX2 protein expression corresponding to panels (H), normalized to β-actin and presented as relative expression levels. Data are presented as mean ± S.D. from n = 3 independent experiments. Statistical significance was assessed by one-way ANOVA followed by Tukey's multiple-comparisons test. *P < 0.05, **P < 0.01, ***P < 0.001 vs. NC group; #P < 0.05, ##P < 0.01, ###P < 0.001 vs. PnAC4A group; ^P < 0.05, ^^P < 0.01, ^^^P < 0.001 vs. RLX group.

We next investigated osteoclast precursor responses using primary BMMs (Fig. 4). Cell-cycle profiling again demonstrated preserved phase distributions under normoxia across treatment groups, indicating no cell-cycle blockade by PnAC4A or RLX@PnAC4A (Fig. 4A and B). Apoptosis under normoxia remained comparable among groups (Fig. 4A and C), supporting biocompatibility in osteoclast precursors as well. Under hypoxia, apoptosis was reduced by treatment, with RLX@PnAC4A showing the most prominent decrease relative to controls (Fig. 4D and F), suggesting enhanced cellular resilience in oxygen-deprived conditions. Functionally, osteoclastogenesis was quantified by TRAP staining–based morphometric measurement. Under normoxia, both RLX and RLX@PnAC4A reduced osteoclast formation relative to NC, with free RLX showing a strong suppressive effect (Fig. 4G and H). Under normoxia + LPS, osteoclastogenic activity was further restrained by RLX and RLX@PnAC4A, whereas PnAC4A alone exerted a comparatively modest effect (Fig. 4G and I). Under hypoxia, RLX@PnAC4A achieved the greatest reduction in osteoclast metrics (Fig. 4J and K), and this inhibition was further amplified under hypoxia + LPS, where RLX@PnAC4A consistently outperformed free RLX and PnAC4A (Fig. 4J and L). These data indicate that RLX@PnAC4A suppresses osteoclast differentiation most effectively under hypoxic and inflammatory stress.

**Fig. 4:**
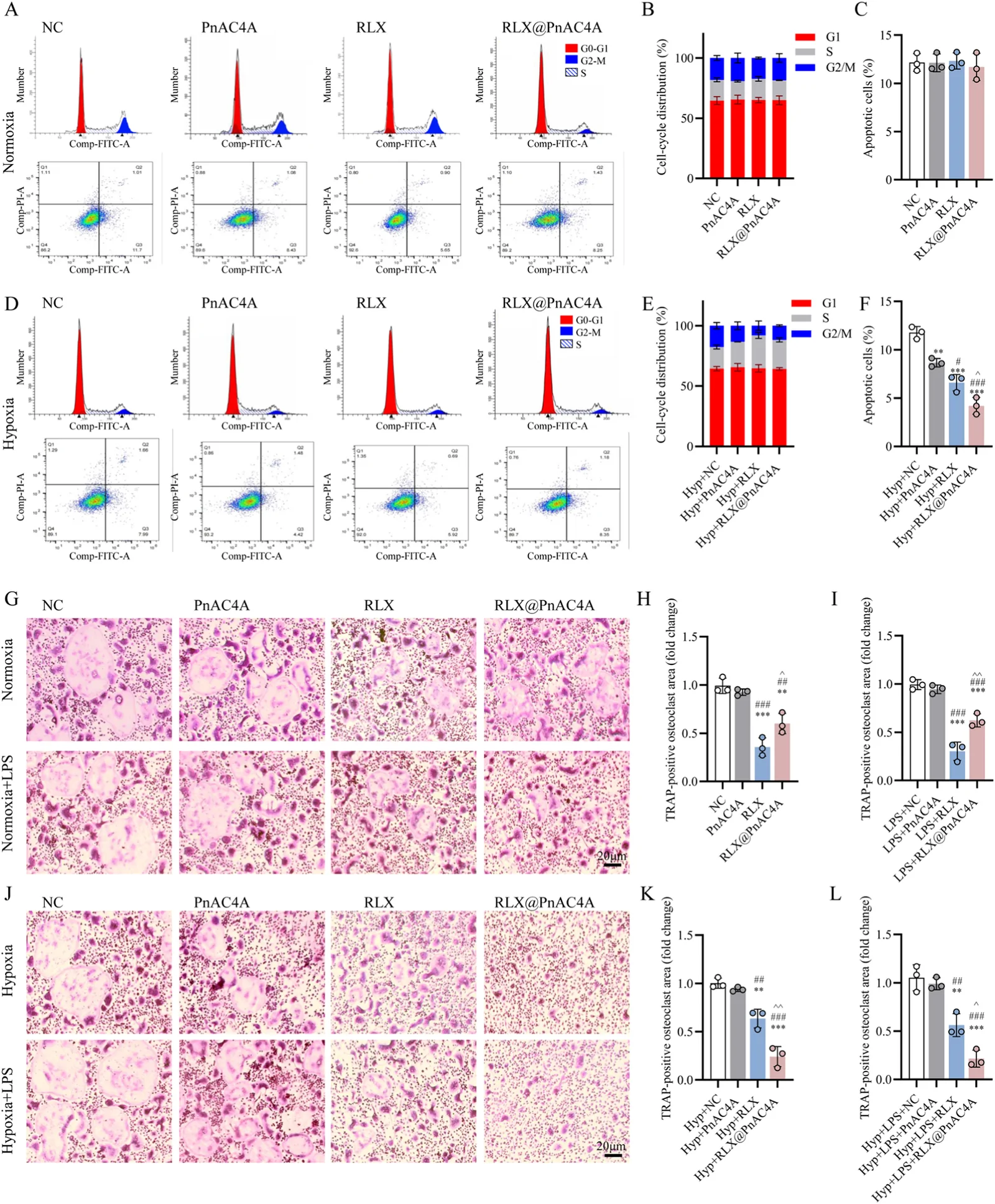
**RLX@PnAC4A inhibits osteoclastogenesis with maximal efficacy under hypoxia and hypoxic inflammation.** (A) Representative flow-cytometry profiles of bone marrow–derived macrophages (primary BMMs) cultured under normoxia and treated with NC, PnAC4A, RLX, or RLX@PnAC4A. Upper panels: DNA-content histograms for cell-cycle analysis; lower panels: Annexin V/PI dot plots for apoptosis assessment. (B) Quantification of cell-cycle phase distribution (G1, S, and G2/M) under normoxia corresponding to (A). (C) Quantification of apoptotic rate under normoxia corresponding to (A). (D) Representative flow-cytometry profiles of primary BMMs cultured under hypoxia following the indicated treatments. Upper panels: DNA-content histograms; lower panels: Annexin V/PI dot plots. (E) Quantification of cell-cycle phase distribution under hypoxia corresponding to (D). (F) Quantification of apoptotic rate under hypoxia corresponding to (D). (G) Representative TRAP staining images showing osteoclast differentiation under normoxia (top row) and normoxia + LPS (bottom row) following treatment with NC, PnAC4A, RLX, or RLX@PnAC4A. (H, I) Quantification of osteoclast differentiation expressed as average OC area (fold change) under normoxia (H) and normoxia + LPS (I), corresponding to (G). (J) Representative TRAP staining images under hypoxia (top row) and hypoxia + LPS (bottom row) after the indicated treatments. (K, L) Quantification of average OC area (fold change) under hypoxia (K) and hypoxia + LPS (L) corresponding to (J). Data are presented as mean ± S.D. from n = 3 independent experiments. Statistical significance was assessed by one-way ANOVA followed by Tukey's multiple-comparisons test. *P < 0.05, **P < 0.01, ***P < 0.001 vs. NC group; #P < 0.05, ##P < 0.01, ###P < 0.001 vs. PnAC4A group; ^P < 0.05, ^^P < 0.01, ^^^P < 0.001 vs. RLX group.

To further determine whether RLX@PnAC4A affected the cytoskeletal polarization and structural maturation of osteoclasts, F-actin organization was examined using fluorescent phalloidin staining. Under vehicle-treated conditions, RANKL-induced BMM-derived osteoclasts displayed large multinucleated morphology accompanied by prominent peripheral F-actin rings, a characteristic structural feature of functionally mature osteoclasts (Supplementary Fig. S7A). PnAC4A alone exerted only a limited effect on F-actin ring formation, whereas free RLX reduced the number of F-actin ring-positive multinucleated osteoclasts. RLX@PnAC4A markedly disrupted the formation of large, continuous F-actin rings under hypoxic and combined hypoxic/inflammatory conditions (Supplementary Fig. S7A–E). Quantitative analysis confirmed a substantial reduction in F-actin ring-positive osteoclasts following RLX@PnAC4A treatment. Notably, under normoxic conditions, free RLX produced a stronger reduction in F-actin ring formation than RLX@PnAC4A. In contrast, the inhibitory effect of RLX@PnAC4A was enhanced under hypoxia, consistent with hypoxia-responsive RLX release from the supramolecular carrier. Together with the reduced bone-resorption area, these results indicate that RLX@PnAC4A suppresses not only osteoclast differentiation but also the cytoskeletal polarization and functional maturation of osteoclasts.

To investigate whether the inhibition of osteoclast formation was accompanied by suppression of the osteoclastogenic protein program, NFATc1 and CTSK protein abundance was examined in RANKL-induced primary BMM-derived osteoclasts. NFATc1 functions as a central regulator of osteoclast differentiation, whereas CTSK is a major proteolytic effector associated with the bone-resorptive phenotype of mature osteoclasts. Western blot analysis showed that PnAC4A alone exerted relatively limited effects on NFATc1 and CTSK expression, whereas free RLX reduced both proteins under the examined environmental conditions (Supplementary Fig. S8A–I). RLX@PnAC4A produced marked reductions in NFATc1 and CTSK under hypoxia and combined hypoxic/inflammatory stress. In particular, the lowest protein abundance was observed following RLX@PnAC4A treatment under the hypoxia plus LPS condition. Under normoxic conditions, free RLX generally reduced NFATc1 and CTSK more strongly than RLX@PnAC4A. In contrast, the inhibitory activity of RLX@PnAC4A became more pronounced under hypoxic conditions, an environment-dependent pattern consistent with enhanced payload release following cleavage of the hypoxia-responsive azo linkage. These results indicate that RLX@PnAC4A suppresses both the differentiation-associated and resorption-associated protein programs of osteoclasts under hypoxic and inflammatory stress.

To determine whether the observed inhibition of osteoclast differentiation translated into reduced bone-resorbing function, mature osteoclasts were cultured on bone slices, and resorption lacunae were evaluated by scanning electron microscopy (SEM). Under untreated and inflammatory conditions, characteristic resorption pits were readily observed on the bone surfaces, whereas RLX@PnAC4A treatment markedly reduced pit formation. Quantitative analysis of the resorbed surface area confirmed that RLX@PnAC4A significantly suppressed osteoclast-mediated bone resorption under normoxic, inflammatory, hypoxic, and hypoxic inflammatory conditions. PnAC4A alone also produced a moderate reduction in resorption activity under selected stress conditions, consistent with its carrier-associated redox-regulatory effect; however, RLX@PnAC4A generally exerted the strongest inhibitory effect. These results provide direct functional evidence that RLX@PnAC4A suppresses not only osteoclast differentiation but also the terminal bone-resorbing activity of mature osteoclasts (Supplementary Fig. S3).

Collectively, these results establish a coherent, bidirectional remodeling profile. RLX@PnAC4A promotes osteogenic differentiation and matrix mineralization in both MC3T3-E1 cells and primary BMSCs, while suppressing osteoclast differentiation and functional bone resorption, with its therapeutic advantage preferentially manifested under hypoxic and inflammatory conditions.

### 2.3 In vivo therapeutic efficacy of RLX@PnAC4A in an osteoporotic mouse model

To evaluate the skeletal targeting of PnAC4A in vivo, Cy5-labeled PnAC4A was intravenously administered and monitored by whole-body fluorescence imaging (Fig. 5). Skeletal-associated fluorescence was detectable within 1 h and became progressively more prominent in the spine and limbs at 6 and 12 h after injection (Fig. 5A). Representative cross-group and ex vivo images showed greater skeletal retention of Cy5–PnAC4A than that of the non-phosphonated control Cy5–SAC4A or free Cy5 (Fig. 5B and C). To further characterize its temporal biodistribution, the spine, hindlimb bones, and major organs were collected at 1, 6, 12, 24, and 48 h after injection for ex vivo fluorescence imaging and region-of-interest quantification. Fluorescence signals in the spine and hindlimbs progressively increased and remained elevated throughout the 48-h observation period. In contrast, liver and kidney signals increased during the early phase, peaked at approximately 12 h, and subsequently declined, whereas comparatively low fluorescence was detected in the heart, spleen, and lungs (Supplementary Fig. S2). These results indicate time-dependent skeletal accumulation and sustained retention of the Cy5-labeled carrier, accompanied by transient off-target organ distribution. The ROS levels were also assessed in bone-derived cells isolated from treated animals. OVX markedly increased ROS relative to sham controls (Fig. 5D and E). PnAC4A partially attenuated this ROS elevation, whereas RLX@PnAC4A achieved a stronger reduction, lowering ROS toward sham levels (Fig. 5E). These data indicate that bone-targeted delivery translates into functional redox modulation in vivo, with added benefit from the RLX-loaded, microenvironment-responsive formulation.

**Fig. 5:**
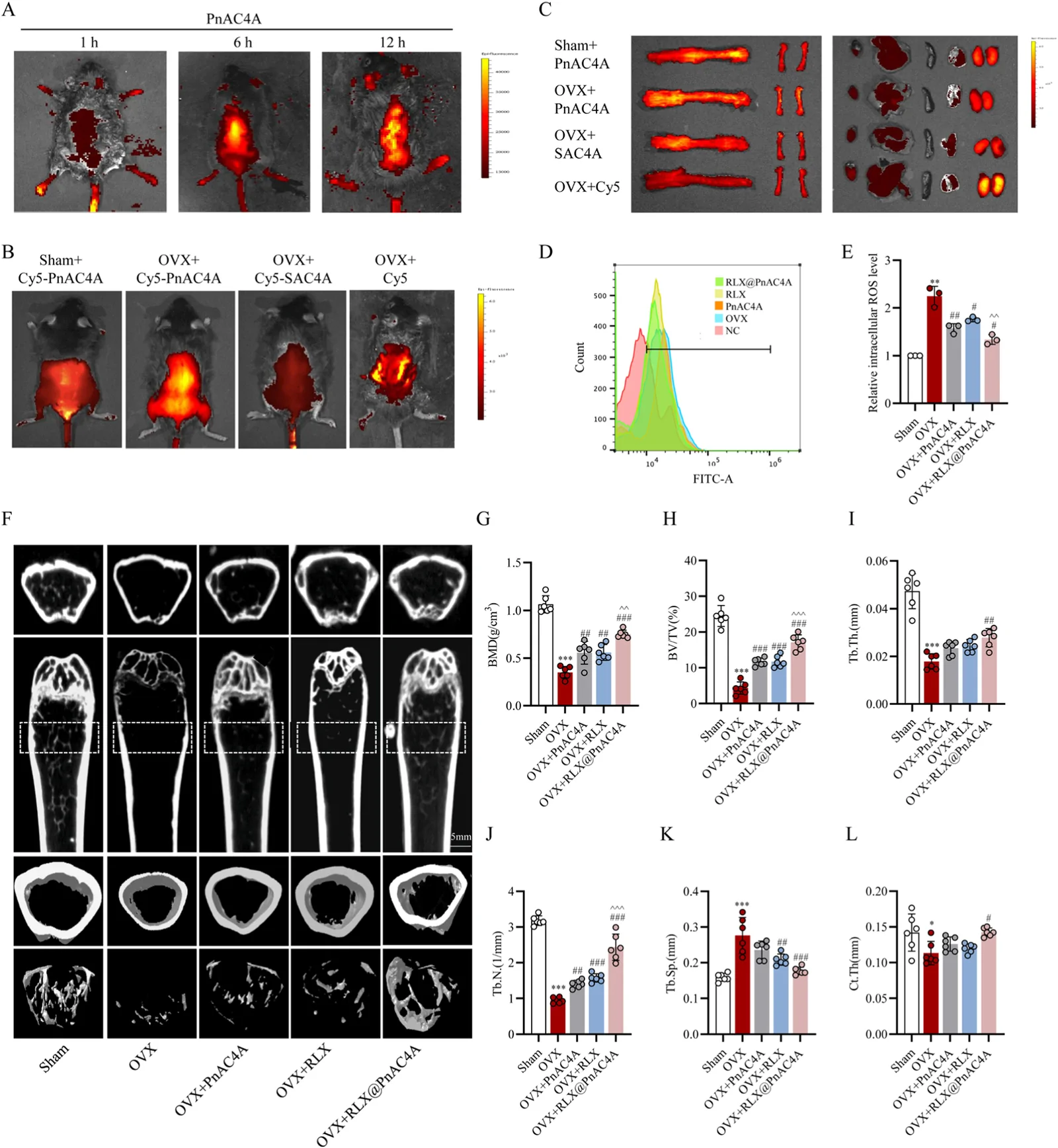
**Bone-targeted accumulation, in vivo redox modulation, and micro-CT–defined bone rescue by RLX@PnAC4A in OVX mice.** (A) Representative whole-body fluorescence images of ovariectomized (OVX) mice at the indicated time points after tail-vein injection of Cy5-labeled PnAC4A, showing progressive enrichment of fluorescence signals in skeletal regions. (B) Cross-group comparison of in vivo fluorescence distribution at the indicated imaging time point, including OVX mice receiving Cy5–PnAC4A, sham-operated mice receiving Cy5–PnAC4A, OVX mice receiving the non-phosphonated control SAC4A, and OVX mice receiving free Cy5. (C) Ex vivo fluorescence imaging of representative bones (femur, tibia, vertebrae) and major organs harvested after in vivo imaging, confirming preferential retention of Cy5–PnAC4A in skeletal tissues relative to controls. (D) Representative flow cytometry profiles of intracellular ROS levels in bone-derived cells isolated from sham and OVX mice treated as indicated (vehicle, PnAC4A, free RLX, or RLX@PnAC4A). (E) Quantification of intracellular ROS levels corresponding to (D), showing OVX-associated ROS elevation and its attenuation by PnAC4A and RLX@PnAC4A. (F) Representative micro-computed tomography (micro-CT) three-dimensional reconstructions of distal femoral trabecular bone from sham, OVX, and treated OVX groups. All micro-CT specimens were acquired, reconstructed, segmented, and displayed using consistent parameters across experimental groups. (G–L) Quantitative micro-CT analysis of trabecular and cortical parameters, including bone mineral density (BMD) (G), bone volume fraction (BV/TV) (H), trabecular thickness (Tb.Th) (I), trabecular number (Tb.N) (J), trabecular separation (Tb.Sp) (K), and cortical thickness (Ct.Th) (L). ROS measurements in (E) were obtained from n = 3 biologically independent mice per group; micro-CT measurements in (G–L) were obtained from n = 6 mice per group. Data are presented as mean ± SD. Statistical significance was assessed by one-way ANOVA followed by Tukey's multiple-comparisons test. *P < 0.05, **P < 0.01, and ***P < 0.001 versus the Sham group; #P < 0.05, ##P < 0.01, and ###P < 0.001 versus the OVX group; ^P < 0.05, ^^P < 0.01, and ^^^P < 0.001 versus the OVX + RLX group.

Micro-CT reconstructions revealed pronounced trabecular deterioration in OVX mice compared with Sham animals, whereas RLX@PnAC4A treatment improved trabecular connectivity and structural integrity (Fig. 5F). Quantitative micro-CT analysis substantiated broad improvements in bone microarchitecture. Compared with vehicle-treated OVX mice, RLX@PnAC4A significantly increased bone mineral density (BMD; Fig. 5G), bone volume fraction (BV/TV; Fig. 5H), trabecular thickness (Tb.Th; Fig. 5I), and trabecular number (Tb.N; Fig. 5J), while reducing trabecular separation (Tb.Sp; Fig. 5K). Cortical thickness was also increased following RLX@PnAC4A treatment (Fig. 5L). To quantify structural normalization, recovery was calculated relative to the OVX-induced deficit. RLX@PnAC4A restored approximately 56% of BMD, 63% of BV/TV, 34% of Tb.Th, and 67% of Tb.N, corrected approximately 85% of the OVX-associated increase in Tb.Sp, and achieved approximately 74% recovery of Ct.Th.

To determine whether structural rescue was accompanied by functional remodeling changes, dynamic histomorphometry and histological/IHC analyses were performed on femoral sections (Fig. 6). Fluorochrome labeling demonstrated reduced inter-label distances in OVX mice, indicative of impaired bone formation, whereas RLX@PnAC4A increased label separation (Fig. 6A), with significant elevations in mineral apposition rate (MAR) and bone formation rate (BFR/BS) relative to OVX controls (Fig. 6B and C). Consistent with these changes, histomorphometric indices of osteoblast activity were reduced by OVX but restored by RLX@PnAC4A, including increases in ObS/BS, N.Ob/BS, and MS/BS (Fig. 6D–F). OVX reduced osteocalcin (OCN) signal, while RLX@PnAC4A increased OCN levels compared with OVX (Fig. 6G). TRAP staining revealed heightened osteoclast activity after OVX that was attenuated by RLX@PnAC4A (Fig. 6A). In line with this, cathepsin K (CTSK) expression was elevated in OVX mice and reduced by RLX and RLX@PnAC4A (Fig. 6A and H). These data demonstrate that RLX@PnAC4A couples bone-selective accumulation with in vivo redox correction, translating into both microarchitectural restoration and a functional shift toward bone formation with restrained resorption in OVX osteoporosis.

**Fig. 6:**
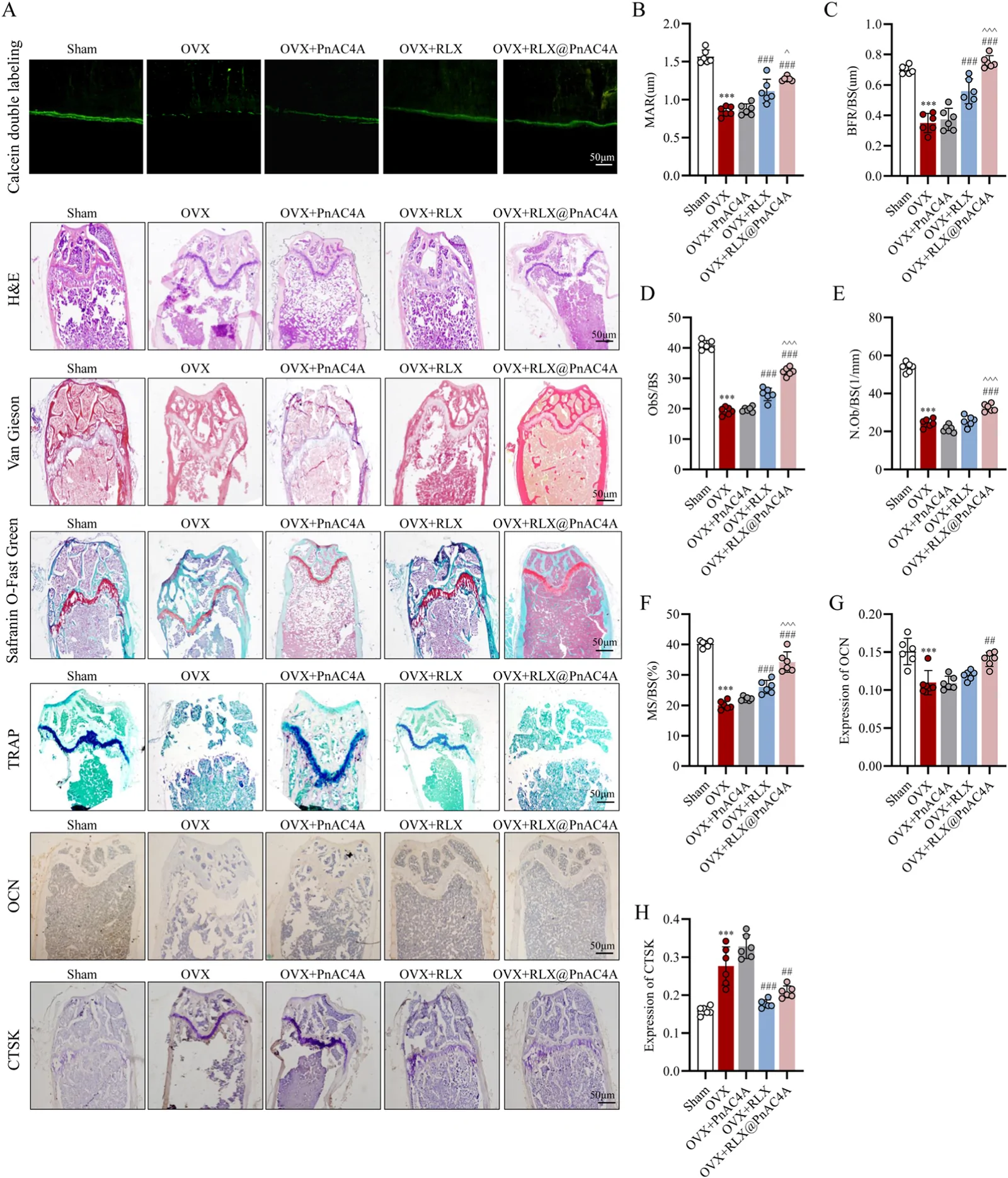
**RLX@PnAC4A restores bone formation dynamics and osteoimmune balance in OVX-induced osteoporosis.** (A) Representative dynamic histomorphometric and histological images of distal femoral sections from sham, OVX, OVX + PnAC4A, OVX + RLX, and OVX + RLX@PnAC4A groups. Top, fluorochrome double labeling (calcein) showing mineral deposition fronts; middle, hematoxylin and eosin (H&E), Van Gieson (VG), and Safranin O–Fast Green (SOFG) staining illustrating trabecular architecture and osteoid deposition; bottom, tartrate-resistant acid phosphatase (TRAP) staining and immunohistochemistry for osteocalcin (OCN) and cathepsin K (CTSK) revealing osteoblast and osteoclast distribution. Within each imaging or staining modality, representative images were acquired from anatomically matched regions at the same magnification using standardized imaging settings and were processed consistently across all experimental groups. (B–C) Quantification of dynamic bone formation parameters, including mineral apposition rate (MAR) and bone formation rate per bone surface (BFR/BS), showing marked enhancement in RLX@PnAC4A-treated mice relative to OVX controls. (D–F) Histomorphometric indices of osteoblast activity—osteoblast surface per bone surface (ObS/BS), osteoblast number per bone surface (N.Ob/BS), and mineralizing surface per bone surface (MS/BS)—demonstrate restoration of osteoblastic function after RLX@PnAC4A treatment. (G) Quantification of OCN-positive area indicates elevated osteoblast differentiation and matrix formation. (H) Quantification of CTSK-positive area showing suppression of osteoclast activity in RLX@PnAC4A- and RLX-treated mice compared with OVX controls. Data are presented as mean ± S.D. from n = 6 mice/group. Statistical significance was determined by one-way ANOVA followed by Tukey's multiple-comparisons test. *P < 0.05, **P < 0.01, ***P < 0.001 vs. Sham group; #P < 0.05, ##P < 0.01, ###P < 0.001 vs. OVX group; ^P < 0.05, ^^P < 0.01, ^^^P < 0.001 vs. OVX + RLX group.

To assess the systemic tolerability of repeated PnAC4A and RLX@PnAC4A administration, major-organ histology, serum hepatic and renal biochemical indices, and circulating inflammatory cytokines were evaluated after the 6-week treatment period. H&E staining of the heart, liver, spleen, lung, and kidney revealed no apparent treatment-related tissue injury or inflammatory infiltration in the PnAC4A- or RLX@PnAC4A-treated groups (Supplementary Fig. S1A). Consistently, serum AST, ALT, creatinine, and urea levels showed no significant treatment-associated abnormalities, indicating no detectable hepatic or renal toxicity under the present dosing regimen (Supplementary Fig. S1B–E). OVX significantly increased circulating IL-6, IL-17, and TNF-α levels relative to Sham controls. PnAC4A partially attenuated these increases, whereas RLX@PnAC4A produced a more pronounced reduction in all three cytokines (Supplementary Fig. S1F–H). Thus, repeated administration of PnAC4A did not induce systemic inflammatory activation, and the RLX-loaded formulation additionally alleviated OVX-associated inflammation. Serum CTX-I was elevated and P1NP was reduced following OVX, whereas RLX@PnAC4A shifted both markers toward the Sham state, consistent with suppression of bone resorption and restoration of bone formation (Supplementary Fig. S1I–J).

### 2.4 Transcriptomic profiling reveals RLX@PnAC4A-mediated remodeling of matrix-associated and osteoimmune programs

To elucidate the molecular programs underlying the therapeutic benefit of RLX@PnAC4A in osteoporotic bone, we performed RNA-seq of bone tissues from Sham, OVX, OVX + PnAC4A, and OVX + RLX@PnAC4A groups and analyzed disease-, material-, and therapy-associated transcriptional states (Fig. 7, Fig. 8).

**Fig. 7:**
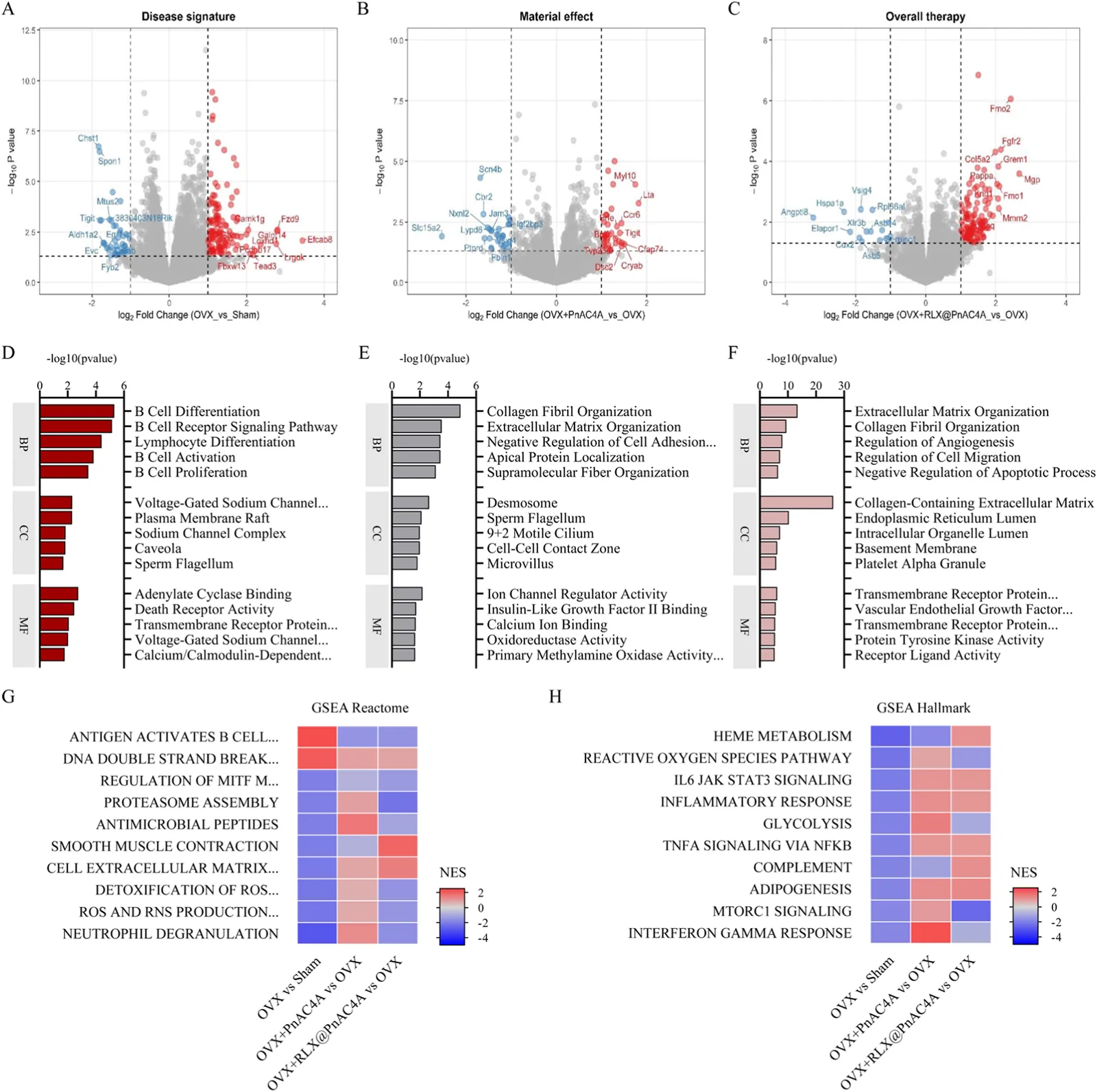
**Transcriptomic dissection of disease-, material-, and therapy-associated transcriptional programs in osteoporotic bone.** (A–C) Volcano plots showing differentially expressed genes (DEGs) in bone tissues across three pairwise comparisons: (A) disease signature (OVX vs. Sham), (B) material effect (OVX + PnAC4A vs. OVX), and (C) overall therapeutic effect (OVX + RLX@PnAC4A vs. OVX). Red and blue dots indicate significantly upregulated and downregulated genes, respectively (|log_2_ fold change| ≥ threshold, adjusted p < 0.05). Numbers denote total DEGs in each direction. Representative genes are annotated. (D–F) Gene Ontology (GO) enrichment analysis of DEGs corresponding to the three transcriptional states. (G) Gene set enrichment analysis (GSEA) based on Reactome pathways was performed on the disease contrast (OVX vs. Sham). Heatmap shows normalized enrichment scores (NES) of representative pathways across OVX vs. Sham, OVX + PnAC4A vs. OVX, and OVX + RLX@PnAC4A vs. OVX comparisons. (H) GSEA heatmap of Hallmark gene sets derived from the disease contrast (OVX vs. Sham).

**Fig. 8:**
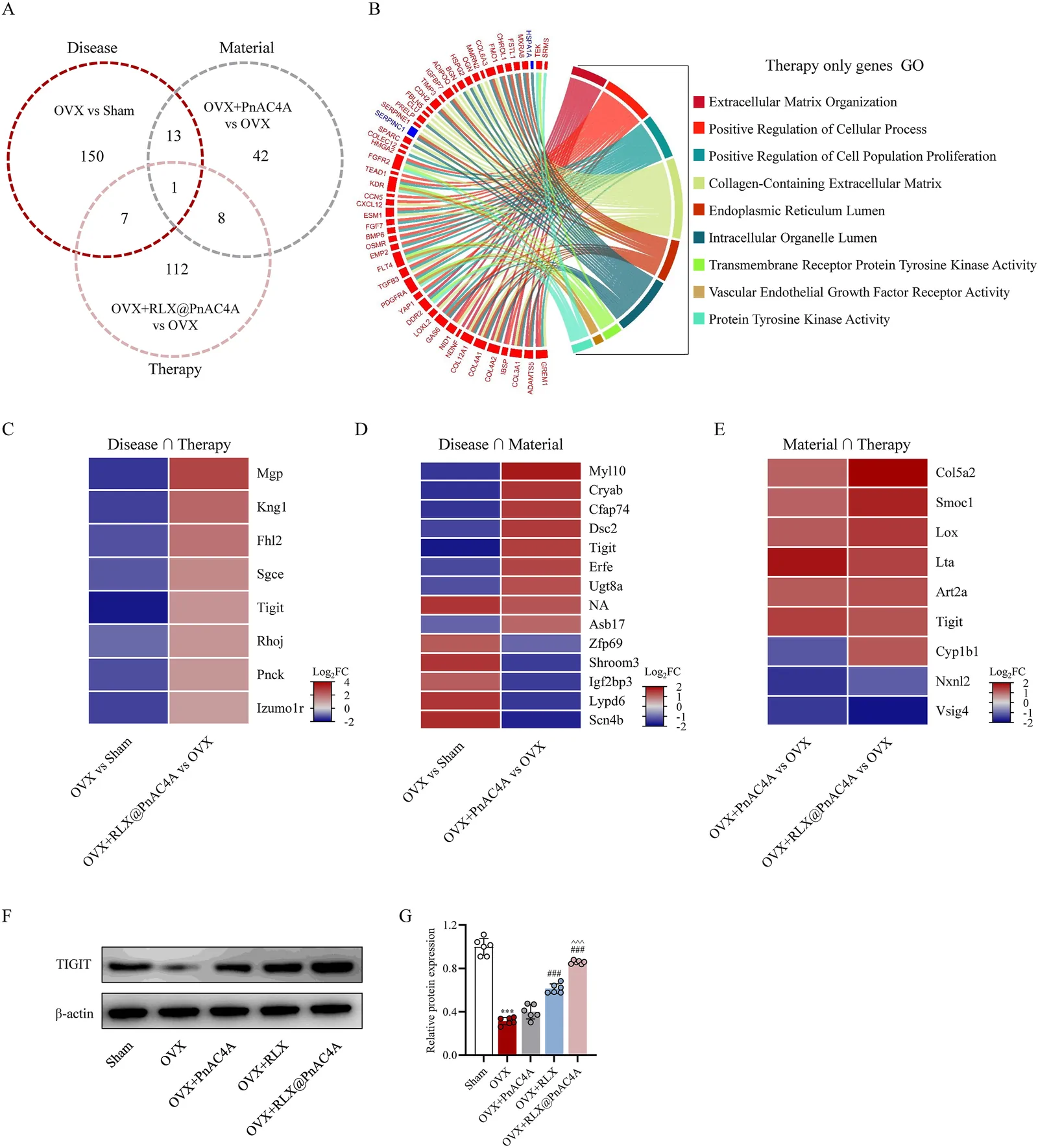
**Integrated transcriptomic analysis delineates therapy-specific gene modules underlying RLX@PnAC4A efficacy.** (A) Venn diagram illustrating the overlap of differentially expressed genes (DEGs) among disease (OVX vs. Sham), material (OVX + PnAC4A vs. OVX), and therapy (OVX + RLX@PnAC4A vs. OVX) comparisons. The majority of DEGs induced by RLX@PnAC4A (n = 112) are uniquely present in the therapy contrast, indicating the emergence of a transcriptional program that is not a linear extension of either disease reversal or material effect. (B) Circular GO enrichment plot of therapy-specific genes (n = 112) uniquely altered in the OVX + RLX@PnAC4A vs. OVX comparison. (C) Heatmap showing genes shared between disease and therapy contrasts. (D) Heatmap of genes overlapping between disease and material contrasts, reflecting partial transcriptional correction by PnAC4A alone. (E) Heatmap of genes shared between material and therapy contrasts. (F) Representative Western blot images of TIGIT and β-actin in bone tissues from Sham, OVX, OVX + PnAC4A, OVX + RLX, and OVX + RLX@PnAC4A mice. (G) Densitometric quantification of TIGIT protein abundance normalized to β-actin. Each dot represents one individual mouse. Data are presented as mean ± S.D. from n = 6 mice per group. *P < 0.05, **P < 0.01, ***P < 0.001 versus Sham; #P < 0.05, ##P < 0.01, ###P < 0.001 versus OVX; ^P < 0.05, ^^P < 0.01, ^^^P < 0.001 versus OVX + RLX.

Differential expression analysis demonstrated a prominent OVX-driven transcriptional shift. In the disease comparison (OVX vs Sham), volcano plotting revealed 129 upregulated and 41 downregulated genes (Fig. 7A), indicating that estrogen deprivation induces broad transcriptional reprogramming in bone. GO enrichment of the disease signature highlighted a strong B-cell–associated immune activation, including B cell differentiation, B cell receptor signaling pathway, lymphocyte differentiation, B cell activation, and B cell proliferation (Fig. 7D), supporting an osteoimmune-perturbed microenvironment in OVX bone.

In contrast to the disease signature, the material comparison (OVX + PnAC4A vs OVX) exhibited a more selective transcriptional footprint, with 35 upregulated and 29 downregulated genes (Fig. 7B). Functional annotation of these material-responsive genes preferentially mapped to structural remodeling processes, most prominently collagen fibril organization, extracellular matrix organization, and related terms linked to cell–matrix architecture (Fig. 7E). The overall therapeutic comparison (OVX + RLX@PnAC4A vs OVX) revealed a stronger transcriptional response, with 114 upregulated and 14 downregulated genes (Fig. 7C). GO enrichment showed a coordinated therapeutic program dominated by extracellular matrix organization and collagen fibril organization, coupled to regulatory modules including regulation of angiogenesis, regulation of cell migration, and negative regulation of the apoptotic process (Fig. 7F).

To examine coordinated pathway perturbations associated with osteoporosis, GSEA was performed for the disease contrast (OVX vs Sham). The top enriched Reactome and Hallmark gene sets defining the OVX state are shown in Fig. 7G–H. In Reactome GSEA, OVX bone showed positive enrichment of immune-related programs such as antigen-activated B-cell signaling, which was reversed to negative NES in both PnAC4A and RLX@PnAC4A groups. Conversely, pathways associated with tissue structure and remodeling, including cell/extracellular matrix–related processes and smooth muscle contraction, were negatively enriched in OVX but shifted toward positive NES, with the strongest inversion observed in the RLX@PnAC4A group (Fig. 7G). Consistently, Hallmark GSEA revealed that multiple stress- and inflammation-related pathways, including IL6–JAK–STAT3 signaling, TNFα signaling via NF-κB, and inflammatory response, were negatively enriched in OVX relative to Sham but became positively enriched following PnAC4A or RLX@PnAC4A treatment. In contrast, metabolic and redox-associated programs such as the reactive oxygen species pathway and glycolysis displayed selective NES inversion primarily in the PnAC4A group, whereas heme metabolism and complement showed therapy-specific inversion in RLX@PnAC4A (Fig. 7H).

Because bulk RNA-seq from whole bone tissue represents signals from multiple cellular populations, we further evaluated whether treatment-associated transcriptional alterations were accompanied by changes in cellular composition. CIBERSORTx-based deconvolution analysis was performed using a customized signature matrix to estimate the relative abundance of major bone-associated populations, including osteoblasts, osteoclasts, neutrophils, monocytes, dendritic cells, T cells, and B cells (Supplementary Fig. S6). The inferred cellular landscape demonstrated distinct remodeling patterns among experimental groups. OVX mice exhibited altered distributions of bone-related and immune populations compared with Sham controls, whereas RLX@PnAC4A treatment partially reversed these alterations. In particular, RLX@PnAC4A reduced the estimated osteoclast-associated fraction and modified immune cell composition toward a pattern closer to the Sham group. These findings suggest that RLX@PnAC4A regulates osteoporotic bone repair through coordinated modulation of cellular composition and the transcriptional microenvironment.

### 2.5 Integrated transcriptomic analysis delineates therapy-specific gene modules underlying RLX@PnAC4A efficacy

To further delineate how material conditioning and drug delivery are integrated at the transcriptional level, we compared differential gene sets derived from disease (OVX vs Sham), material (OVX + PnAC4A vs OVX), and therapy (OVX + RLX@PnAC4A vs OVX) contrasts (Fig. 8A). Venn analysis revealed limited overlap among these three states, indicating that RLX@PnAC4A induces a transcriptional program that is not a simple linear extension of either disease reversal or material effect.

Notably, the therapy comparison yielded 112 genes uniquely altered only in the OVX + RLX@PnAC4A vs OVX contrast, with no overlap with either the disease- or material-responsive gene sets (Fig. 8A). Functional annotation of these therapy-specific genes revealed strong enrichment in pathways associated with extracellular matrix organization, collagen-containing extracellular matrix, positive regulation of cellular processes, and positive regulation of cell population proliferation, as well as signaling categories related to transmembrane receptor protein tyrosine kinase activity and vascular endothelial growth factor receptor activity (Fig. 8B). These findings indicate that RLX loading activates a distinct transcriptional module centered on matrix remodeling and pro-regenerative signaling that is not elicited by the carrier alone.

In contrast, a small subset of genes overlapped between disease and therapy (Fig. 8C), including Mgp, Kng1, Fhl2, Sgce, Tigit, Rhoj, Pnck, and Izumo1r, which displayed opposite expression trends between OVX and RLX@PnAC4A-treated groups. These genes span immune regulation, vascular–stromal interaction, and cytoskeletal organization, indicating node-level transcriptional rescue of disease-associated dysregulation. The disease material overlap comprised 13 genes (Fig. 8D), including Myl10, Cryab, Cfap74, Dsc2, Tigit, Erfe, Ugt8a, and Igf2bp3, reflecting partial correction of OVX-induced transcriptional changes by PnAC4A alone at selected structural, stress-responsive, and post-transcriptional regulatory nodes. Finally, analysis of the material–therapy overlap identified 8 shared genes (Fig. 8E), including Col5a2, Smoc1, Lox, Lta, Art2a, Tigit, Cyp1b1, Nxnl2, and Vsig4. Most of these genes exhibited concordant regulation under material and therapy conditions, suggesting that PnAC4A establishes a stable transcriptional baseline, particularly in extracellular matrix and immune-associated pathways, that is largely preserved upon RLX loading. In contrast, Cyp1b1 showed opposite regulation between material and therapy groups, indicating a payload-sensitive metabolic node selectively reprogrammed by RLX@PnAC4A.

Notably, Tigit was the only gene shared across all three comparisons, including disease (OVX vs Sham), material (OVX + PnAC4A vs OVX), and therapy (OVX + RLX@PnAC4A vs OVX) (Fig. 8C–E). Tigit transcript abundance was reduced in OVX bone relative to Sham, partially restored by PnAC4A, and further increased following RLX@PnAC4A treatment. To determine whether this transcriptional pattern was reflected at the protein level, TIGIT abundance was examined by Western blotting of whole bone lysates. Consistent with the RNA-seq findings, OVX markedly reduced TIGIT protein levels, whereas PnAC4A partially restored TIGIT expression. Free RLX and RLX@PnAC4A produced a more pronounced recovery, with the RLX@PnAC4A group approaching the Sham level (Fig. 8F and G).

Collectively, these findings support a treatment-associated transcriptional architecture characterized by partial restoration of disease-related signals, material-conditioned remodeling, and activation of a broader therapy-associated module enriched in extracellular matrix organization and regenerative signaling.

## 3 Discussion

The present study establishes RLX@PnAC4A as a hypoxia-responsive, bone-targeted supramolecular therapeutic designed to couple skeletal enrichment with pathology-associated payload release for the treatment of postmenopausal osteoporosis (PMOP). Rather than functioning solely as a passive drug carrier, RLX@PnAC4A operates through three interconnected levels of activity: phosphonate-mediated anchoring promotes preferential accumulation at mineralized bone surfaces; cleavage of the azo-containing supramolecular structure under low-oxygen and reductive conditions facilitates local RLX release; and the combined carrier–payload system modulates redox imbalance, osteoblast–osteoclast coupling, and the tissue-level remodeling environment. This framework provides a coherent explanation for the enhanced activity of the formulation under hypoxic and inflammatory stress, while avoiding attribution of the therapeutic effect to a single molecular pathway.

Calixarenes represent a versatile family of macrocyclic hosts whose structurally defined cavities and modular functionalization enable programmable host–guest binding and stimulus-responsive behavior [27,28]. In the present system, phosphonate decoration conferred strong affinity for hydroxyapatite and promoted time-dependent skeletal enrichment, consistent with the established mineral-binding properties of phosphonate and bisphosphonate motifs [29,30]. The greater bone-associated fluorescence observed with PnAC4A than with the non-phosphonated control supports an important contribution of chemical anchoring, rather than nonspecific distribution alone, to skeletal localization. In estrogen-deficient bone, this affinity may be particularly relevant at high-turnover trabecular remodeling surfaces, where exposed mineral interfaces provide binding opportunities for phosphonate-containing ligands [31]. Time-resolved biodistribution further showed a progressive decline in carrier-associated fluorescence in major non-skeletal organs, whereas skeletal signals remained comparatively sustained over the observation period. These findings support preferential skeletal retention and reduced non-skeletal exposure, although fluorescence imaging alone does not establish complete clearance, degradation products, or specific elimination pathways.

Importantly, because hypoxia and inflammation are not unique to skeletal tissue, the bone affinity of RLX@PnAC4A should not be attributed to hypoxia responsiveness alone. Instead, the formulation operates through sequential skeletal localization and microenvironment-responsive activation. Multivalent phosphonate–hydroxyapatite interactions promote preferential adsorption and retention at mineralized bone surfaces, whereas reduction of the azo linkages under hypoxia-associated reductive conditions is designed to destabilize the host–guest assembly and facilitate payload release after localization. Accordingly, RLX@PnAC4A provides preferential rather than absolute skeletal specificity, and off-target release remains theoretically possible in non-skeletal tissues where sufficient carrier exposure coincides with a reducing microenvironment.

Estrogen deficiency is accompanied by vascular, metabolic, inflammatory, and redox alterations within bone marrow and trabecular compartments, potentially generating local regions with impaired oxygen delivery and increased inflammatory signaling [32]. Hypoxia-associated immune regulation, including alterations involving B-lineage compartments, has also been implicated in estrogen-deficiency-related bone loss, whereas specialized skeletal vascular subtypes are closely linked to osteogenesis and are disrupted in bone disease [33,34]. Against this background, the combined hypoxia and LPS condition used in the present study was designed as a controlled dual-stress model incorporating reduced oxygen availability and TLR4-mediated inflammatory stimulation. It was not intended to reproduce the full complexity of the PMOP microenvironment. In the osteoclast experiments, RANKL remained the essential differentiation stimulus, and LPS was introduced after RANKL-mediated lineage priming to impose inflammatory stress on committed osteoclast precursors. This distinction is important because the effects of LPS on osteoclastogenesis depend on the differentiation stage, concentration, and timing of exposure. Accordingly, the present experiments were primarily designed to evaluate whether RLX@PnAC4A retained or enhanced activity under defined hypoxic and inflammatory conditions rather than to establish that LPS and hypoxia exert a universal synergistic effect on osteoclastogenesis.

A central feature of RLX@PnAC4A is the integration of microenvironment-responsive release with redox regulation. Excessive reactive oxygen species impair osteoblast differentiation and survival, amplify inflammatory signaling, and facilitate osteoclastogenesis, making oxidative imbalance an important component of osteoporosis pathogenesis [35]. In the present study, PnAC4A alone partially reduced ROS accumulation under hypoxic and inflammatory stress, suggesting a carrier-associated microenvironmental effect. RLX loading further enhanced this response, particularly under low-oxygen conditions in which activation of the azo-containing supramolecular system would be expected to facilitate payload release. The accompanying restoration of osteogenic mineralization and suppression of osteoclast formation therefore support redox modulation as one functional link between environment-responsive drug delivery and rebalanced bone remodeling. Nevertheless, because upstream redox sensors were not directly manipulated, the present findings support an association between ROS attenuation and therapeutic activity rather than establishing ROS as the sole causal mediator.

At the osteoclast level, the newly added molecular, structural, and functional analyses further define the anti-resorptive activity of RLX@PnAC4A. Western blotting showed that RLX@PnAC4A reduced the abundance of NFATc1 and CTSK in RANKL-induced primary BMM-derived osteoclasts, with particularly pronounced effects under hypoxia and combined hypoxic/inflammatory stress. NFATc1 is a central transcriptional regulator of osteoclast differentiation, whereas CTSK is a major proteolytic effector associated with the matrix-resorptive phenotype of mature osteoclasts. Their coordinated reduction therefore indicates suppression of both the differentiation-associated osteoclastogenic program and the acquisition of resorption-associated activity. Consistent with these molecular changes, RLX@PnAC4A disrupted the organization of peripheral F-actin rings, which are required for osteoclast polarization and formation of the specialized resorptive compartment. This structural impairment was accompanied by a marked reduction in resorption lacunae on bovine bone slices. The bone-slice assay is particularly important because it demonstrates direct suppression of matrix-resorptive function rather than merely a reduction in TRAP positivity or osteoclast-marker expression. Together, the decreases in NFATc1 and CTSK, disruption of F-actin ring organization, and reduction in bone-slice resorption provide convergent molecular, structural, and functional evidence that RLX@PnAC4A interferes with osteoclast differentiation and functional maturation. However, because NFATc1 and CTSK were not directly manipulated, they should be interpreted as treatment-responsive components of the osteoclastogenic program rather than direct molecular targets of RLX@PnAC4A.

These findings are consistent with recent pharmacological studies showing that effective suppression of osteoporotic bone loss requires intervention not only in osteoclast formation but also in the structural and resorptive functions of mature osteoclasts. Tetrahydroberberine was reported to suppress RANKL-induced osteoclast formation and bone resorption through attenuation of MAPK and NF-κB signaling, while also promoting apoptosis in mature osteoclasts and improving skeletal microarchitecture in OVX mice [36]. Idebenone similarly inhibited osteoclast differentiation by reducing oxidative stress and suppressing AKT/MAPK signaling; importantly, its anti-resorptive activity was supported by F-actin ring staining, resorption-pit analysis, protein-level validation, and improvement of bone mass in OVX mice [37]. These studies reinforce the value of assessing osteoclast activity at multiple biological levels. In the present study, the combined NFATc1/CTSK, F-actin, and bone-slice data provide a similarly integrated assessment of osteoclast differentiation and function. However, MAPK, NF-κB, and AKT signaling were not directly examined here, and these pathways therefore cannot be assigned as the specific intracellular mechanism of RLX@PnAC4A action.

Consistent with the cellular and functional findings, transcriptomic profiling of whole bone tissue revealed prominent immune-associated remodeling in OVX mice, including enrichment of B-cell-related programs. This observation is compatible with evidence that B-lineage populations can contribute osteoclastogenic signals and immune dysregulation under estrogen-deficient conditions [34,38]. However, bulk bone RNA-seq integrates transcriptional signals from osteoblast-lineage cells, osteoclast-lineage cells, stromal populations, vascular cells, and multiple immune compartments. The observed pathway changes therefore cannot be assigned directly to a single cell population. To assess the potential contribution of cellular composition, CIBERSORTx-based deconvolution was performed using a customized reference signature matrix. The inferred profiles suggested group-dependent changes in the relative distribution of modeled bone-related and immune populations. These results indicate that the bulk transcriptomic signatures likely reflect an integrated combination of cellular-composition remodeling and transcriptional responses within the tissue microenvironment. Computational deconvolution does not, however, provide definitive cell-specific localization or distinguish all within-lineage expression changes from changes in cell abundance.

Within this tissue-level framework, PnAC4A partially reversed disease-associated transcriptional alterations and preferentially shifted enriched programs toward extracellular-matrix organization and cytoskeletal remodeling, changes compatible with restoration of a more permissive structural environment. RLX@PnAC4A was associated with a broader transcriptional response involving ECM remodeling, receptor-linked signaling, and vascular/angiogenic programs, which are relevant to osteoblast–matrix interactions, vascularized osteogenesis, and restoration of bone homeostasis [39]. These pathway-level findings, together with the inferred changes in cellular composition, support coordinated remodeling of the osteoimmune and matrix-regulatory environment. They do not, however, establish whether each pathway change arose predominantly from altered cell abundance, transcriptional regulation within specific lineages, or a combination of both mechanisms.

Integrative comparison of disease-, carrier-, and therapy-associated transcriptomic changes identified Tigit as the only gene shared across all three comparisons. Tigit transcript abundance was reduced in OVX bone, partially restored by PnAC4A, and further increased following RLX@PnAC4A treatment. Western blotting of whole-bone lysates confirmed a directionally concordant change in TIGIT protein abundance, supporting TIGIT as a tissue-level marker associated with osteoporosis and treatment response. Nevertheless, neither bulk RNA-seq nor whole-bone Western blotting can identify the specific cellular source of TIGIT. The observed difference may reflect changes in immune-cell abundance, activation state, cell-intrinsic expression, or a combination of these factors. Moreover, TIGIT activity was not directly manipulated in the present study. TIGIT should therefore be interpreted as a convergent tissue-level marker associated with treatment-responsive osteoimmune remodeling rather than a demonstrated mechanistic mediator or validated therapeutic target. Cell-resolved localization and functional perturbation will be required to determine whether TIGIT actively contributes to bone remodeling or primarily reflects changes in the immune composition of the osteoporotic niche.

The tissue-level response observed here is consistent with emerging biomaterial strategies that combine skeletal targeting with pathology-responsive payload delivery. A recently developed bone-targeting ROS-responsive nanoplatform, PH/DPA@A, integrated an Asp8-containing bone-affinitive ligand with a ROS-cleavable hyaluronic-acid-based shell to deliver the monocarboxylate-transporter inhibitor AZD3965. By blocking lactate transport and suppressing osteoclast glycolytic metabolism, the system reduced osteoclast formation and resorptive activity and improved trabecular bone parameters in OVX mice [40]. This platform and RLX@PnAC4A share the broader design principle of coupling bone affinity with disease-responsive drug release, but their molecular architectures and therapeutic strategies differ. PH/DPA@A uses Asp8-mediated skeletal affinity and ROS-responsive release to inhibit osteoclast glycolysis, whereas RLX@PnAC4A employs phosphonate-mediated mineral anchoring and hypoxia-responsive destabilization of a calixarene host–guest system to deliver raloxifene. In addition to suppressing osteoclast differentiation and resorption, RLX@PnAC4A was evaluated for effects on osteoblast mineralization, redox balance, and tissue-level ECM, vascular, and osteoimmune remodeling. These distinctions position the present system as a complementary rather than directly comparable strategy for systemic PMOP treatment.

Other responsive biomaterial approaches have focused primarily on local osteoporotic implant interfaces. A pH-responsive mussel-inspired adhesive improved osteoporotic bone–implant integration through coordinated immune, angiogenic, and osteogenic regulation [17], whereas an integrin α2β1-targeting biomimetic coating enhanced interfacial osseointegration by promoting M2 macrophage polarization and vascularized bone regeneration [18]. Unlike these locally applied implant-interface systems, RLX@PnAC4A is an injectable, bone-seeking supramolecular formulation intended for non-implant-associated PMOP. By combining phosphonate-mediated skeletal enrichment, hypoxia-responsive drug release, and redox-associated remodeling, RLX@PnAC4A extends microenvironment-adaptive biomaterial design from local interface engineering toward systemic pharmacological regulation of osteoporotic bone.

From a formulation perspective, RLX@PnAC4A maintained a relatively stable hydrodynamic diameter and PDI during 36 h of incubation under serum-containing conditions, without a progressive increase suggestive of substantial aggregation. The larger absolute hydrodynamic diameter observed in serum-containing medium than in buffer is compatible with the medium dependence of DLS measurements and should not be interpreted as direct evidence of structural expansion or a defined protein corona. These findings support short-term colloidal stability of the final therapeutic formulation under the tested conditions. Repeated intravenous administration over 6 weeks was not associated with detectable histopathological injury in the heart, liver, spleen, lung, or kidney, and serum indices of hepatic and renal function remained comparable among treatment groups. PnAC4A and RLX@PnAC4A also did not induce systemic inflammatory activation. Instead, serum IL-6, TNF-α, and IL-17 levels were reduced relative to the elevated levels observed in OVX mice, with the loaded formulation producing the more pronounced effect. These results distinguish the absence of material-induced inflammatory toxicity from the additional therapeutic attenuation of OVX-associated systemic inflammation. Collectively, the histological, biochemical, and cytokine data support favorable repeated-dose tolerability under the present 6-week regimen, but they do not constitute a chronic toxicology assessment.

From a translational perspective, RLX@PnAC4A addresses several limitations associated with conventional raloxifene administration, including restricted skeletal selectivity, systemic exposure, and reduced effectiveness within adverse pathological microenvironments [41]. The use of an approved therapeutic payload provides a defined pharmacological basis, whereas the chemically structured calixarene scaffold offers potential advantages in reproducibility and functional modification. Nevertheless, intravenous incorporation of raloxifene into a supramolecular carrier creates a distinct formulation whose pharmacokinetics, tissue exposure, metabolism, and safety cannot be inferred directly from those of orally administered free raloxifene.

Several limitations should therefore be acknowledged. First, only one in vitro RLX-equivalent concentration and one in vivo dose were evaluated. Although these levels were selected from previously reported biologically active ranges [[42], [43], [44], [45], [46]], they were not optimized for the current intravenous formulation. The present findings demonstrate a formulation-associated efficacy advantage at the tested exposure but do not define the minimum effective dose, dose–response relationship, or optimal therapeutic window. Second, the 6-week safety evaluation and 48-h fluorescence-distribution study do not resolve the long-term fate of the PnAC4A scaffold. Quantitative studies are required to determine intact-carrier pharmacokinetics, degradation kinetics, circulating and tissue-associated metabolites, renal and hepatobiliary elimination, hematological effects, and carrier-specific immune responses. Fluorescence decay in non-skeletal organs should be interpreted as declining carrier-associated signal rather than proof of complete material clearance. Third, the combined hypoxia and LPS system represents a simplified dual-stress model rather than the full PMOP microenvironment. More physiologically complex coculture, organoid, or ex vivo bone models will be required to reproduce interactions among osteoblasts, osteoclasts, stromal cells, vascular cells, and immune compartments. Fourth, CIBERSORTx and whole-bone TIGIT Western blotting provide tissue-level inference but cannot resolve cell-specific transcriptional responses or the cellular source of TIGIT. Similarly, the reductions in NFATc1 and CTSK support suppression of the osteoclastogenic program but do not establish either protein as a direct causal target. Future studies incorporating single-cell or spatial transcriptomics, cell-type-specific localization, functional perturbation, proteomics, metabolomics, and pathway-rescue experiments will be required to define the underlying cellular and molecular mechanisms. Extended dose-ranging studies, chronic safety evaluation, and validation in larger animal models will also be necessary before clinical translation.

In summary, this study demonstrates that RLX@PnAC4A functions as a hypoxia-responsive, bone-targeted supramolecular system that improves the local delivery of raloxifene and modulates redox imbalance, osteoblast–osteoclast coupling, and the tissue-level remodeling environment in osteoporotic bone. The coordinated changes in osteogenic mineralization, NFATc1 and CTSK expression, F-actin organization, bone resorption, cellular composition estimates, and bulk-tissue transcriptional programs support a multilevel therapeutic effect. Rather than establishing a single dominant molecular target, the findings position RLX@PnAC4A as a microenvironment-adaptive strategy that integrates material-mediated skeletal localization with payload-dependent regulation of bone remodeling. This work provides a basis for further development of responsive supramolecular systems for metabolic bone disease.

## 4 Materials and methods

### 4.1 Materials and reagents

Raloxifene hydrochloride (RLX, purity ≥98%) was obtained from Sigma–Aldrich (USA). p-tert-Butylcalix [4]arene, 4-hydroxyazobenzene, phosphorus oxychloride (POCl_3_), and all other analytical-grade reagents were purchased from Aladdin Biochemical Co. (Shanghai, China). MC3T3-E1 Subclone 14 mouse calvarial preosteoblasts were purchased from Procell Life Science & Technology Co., Ltd. (Wuhan, China; catalog no. CL-0378). Primary bone marrow mesenchymal stem cells (BMSCs)and Primary bone marrow-derived macrophages (BMMs) were generated from bone marrow cells freshly isolated from the femurs and tibias of 6-week-old female C57BL/6 mice maintained under specific pathogen-free conditions. Cell culture media and supplements were from Gibco (Thermo Fisher Scientific). All aqueous solutions were prepared using ultrapure water (18.2 MΩ cm).

### 4.2 Synthesis and characterization of PnAC4A

Calix [4]arene (C4A) was synthesized according to the reported procedure in the literature. 4-Aminophenylphosphonic acid (216 mg, 1.25 mmol), concentrated hydrochloric acid (2 mL), and distilled water (2 mL) were added to a round-bottom flask. After the mixture became a clear, colorless solution, it was cooled to 0–5 °C. Sodium nitrite (95 mg, 1.38 mmol) was dissolved in distilled water (0.5 mL) and then slowly added dropwise to the round-bottom flask containing the solution at 0–5 °C, generating the diazonium salt. C4A (113 mg, 0.27 mmol), sodium acetate trihydrate (680 mg, 5.00 mmol), methanol (3.5 mL), and DMF (6.0 mL) were added sequentially to a 100 mL round-bottom flask, which was then cooled to 0–5 °C. The diazonium salt was added dropwise to the mixture containing C4A, while maintaining the reaction temperature at 0–5 °C. Pyridine (1 mL) was added, and the reaction was allowed to proceed in an ice bath for 1 h at 0–5 °C, followed by a gradual increase to room temperature. The reaction mixture was then heated to 30 °C and allowed to react for 2 h. Distilled water (30 mL) and concentrated hydrochloric acid (1 mL) were added, and the mixture was heated at 60 °C for 30 min. The reaction solution was concentrated by rotary evaporation under vacuum, and then dialyzed in distilled water using a dialysis bag (MWCO: 500 Da) for 48 h. The remaining solution was concentrated again, and the crude product was recrystallized from methanol and water to afford a dark red product (110 mg, 35% yield). 1H NMR (400 MHz, D_2_O) *δ*: 7.53 – 7.44 (m, 8H), 7.38 (s, 8H), 7.29 (d, *J* = 8.1 Hz, 8H), 4.42 (d, *J* = 12.8 Hz, 4H), 2.94 (d, *J* = 13.2 Hz, 4H).

### 4.3 Preparation of RLX@PnAC4A complex

PnAC4A assemblies were prepared by dissolving the solid in PBS, where they spontaneously self-assemble into supramolecular assemblies. RLX@PnAC4A was prepared by thoroughly mixing weighed solid PnAC4A and RLX in a mortar. The mixture was ground for 30 min and then dissolved in PBS buffer.

### 4.4 Physicochemical characterization

Particle size distribution and zeta potential were measured by dynamic light scattering (DLS; Zetasizer Nano ZS, Malvern). Morphology was observed using transmission electron microscopy (TEM; JEM-2100, JEOL, Japan). The complexation between PnAC4A and RhB was determined by direct fluorescence titration. A mixed solution containing known amounts of the host and guest was successively injected into 2.50 mL of the guest solution in a quartz cuvette. By fitting the fluorescence intensity according to a 1:1 host-guest binding stoichiometry, the association constant was obtained (doi.org/10.31635/ccschem.024.202404596). The excitation (*λ*_ex_) and emission (*λ*_em_) wavelengths of RhB were 554 nm and 575 nm, respectively. The complexation between PnAC4A and RLX was measured by competitive fluorescence titration. A mixed solution containing known amounts of the reporter dye (RhB), the host (PnAC4A), and the competing guest (RLX) was injected into 2.50 mL of a dye–host solution in a quartz cuvette. The fluorescence intensity was recorded before the first addition and after each subsequent addition. The binding constant was obtained by fitting the fluorescence intensity data using a 1:1 competitive binding model [47].

### 4.5 Hypoxia-responsive drug release

The reduction kinetics of PnAC4A were investigated by UV–visible spectroscopy. An excess of SDT (10 mM) was injected into 2.50 mL of a PnAC4A solution (10.0 μM), and the time-dependent absorbance at 420 nm was monitored at 37 °C. The release kinetics of the complex were studied by fluorescence spectroscopy. An excess of SDT (10 mM) was injected into 2.50 mL of a RhB@PnAC4A complex solution (10.0/20.0 μM) in a quartz cuvette, and the time-dependent fluorescence intensity of RhB at 575 nm was monitored at 37 °C.

### 4.6 Cell culture and viability assay

MC3T3-E1 and primary BMMs cells were cultured in α-MEM and DMEM supplemented with 10% FBS and 1% penicillin/streptomycin at 37 °C with 5% CO_2_. To simulate the hypoxic microenvironment of osteoporotic bone, cells were cultured under 1% O_2_, 5% CO_2_, and 94% N_2_ (MCO-5M incubator, Sanyo, Japan). Cell viability following treatment with RLX, PnAC4A, and RLX@PnAC4A (equivalent RLX concentration = 5 μM) was evaluated using the CCK-8 assay after 24 and 48 h incubation. The selected concentration was used as a literature-informed working concentration. Previous studies have demonstrated that raloxifene exerts biological effects in both osteoblast- and osteoclast-lineage cells at low-micromolar concentrations [44,45]. Consistent with these reports, the selected concentration did not measurably reduce cell viability under the experimental conditions used in this study.

### 4.7 Osteogenic differentiation assay

MC3T3-E1 cells and primary mouse BMSCs were seeded in 24-well plates. After cell attachment, osteogenic differentiation was induced using α-MEM supplemented with 10 mM β-glycerophosphate, 50 μg/mL ascorbic acid, and 10 nM dexamethasone. Cells were treated with vehicle control, PnAC4A, free RLX, or RLX@PnAC4A at an equivalent RLX concentration of 5 μM under normoxic or hypoxic conditions, with or without LPS stimulation. Hypoxic cultures were maintained at 1% O_2_, 5% CO_2_, and 94% N_2_. Early osteogenic differentiation was evaluated by alkaline phosphatase staining on day 7 using a commercial ALP staining kit according to the manufacturer's instructions. ALP staining intensity was quantified using ImageJ software and normalized to the corresponding control group. Late-stage matrix mineralization was assessed by Alizarin Red S staining on day 14. After fixation with 4% paraformaldehyde, cells were stained with Alizarin Red S solution and extensively washed with distilled water. For quantitative analysis, the bound dye was dissolved in 10% cetylpyridinium chloride, and absorbance was measured at 570 nm.

### 4.8 Osteoclast differentiation and TRAP staining

Primary bone marrow-derived macrophages were cultured in α-MEM supplemented with 10% fetal bovine serum, 1% penicillin–streptomycin, and M-CSF (50 ng/mL). Osteoclast-lineage differentiation was initiated by treatment with RANKL (100 ng/mL) for 36h. Thereafter, cells were exposed to LPS (100 ng/mL) or an equal volume of vehicle, while M-CSF and RANKL supplementation was maintained. Cells were treated with vehicle control, PnAC4A, free RLX, or RLX@PnAC4A at an equivalent RLX concentration of 5 μM and cultured under normoxia or hypoxia. Normoxic cultures were maintained at 21% O_2_, 5% CO_2_, whereas hypoxic cultures were maintained at 1% O_2_, 5% CO_2_, and 94% N_2_. The four environmental conditions were defined as normoxia, normoxia plus LPS, hypoxia, and hypoxia plus LPS.

### 4.9 F-actin ring staining

Primary BMMs were induced toward osteoclast differentiation using M-CSF and RANKL under the indicated oxygen and inflammatory conditions. Following treatment with vehicle, PnAC4A, free RLX, or RLX@PnAC4A, cells were gently washed three times with phosphate-buffered saline and fixed with 4% paraformaldehyde for 10 min at room temperature. The cells were subsequently washed three times with PBS, permeabilized with 0.1% Triton X-100 in PBS for 5 min at room temperature, and blocked with 2.5% bovine serum albumin in PBS for 20 min. F-actin was stained with Actin-Tracker Red-594 conjugated phalloidin for 1 h at room temperature in the dark. Fluorescence images were acquired using an inverted fluorescence microscope under identical exposure, gain, and acquisition settings for all experimental groups. The number of F-actin ring-positive multinucleated osteoclasts was quantified using ImageJ by an investigator blinded to group allocation.

### 4.10 Osteoclast bone resorption assay

Primary mouse bone marrow-derived macrophages were seeded onto sterile bovine cortical bone slices placed in 96-well plates. Osteoclast differentiation was induced using M-CSF and RANKL in the presence of vehicle, PnAC4A, free RLX, or RLX@PnAC4A at an equivalent RLX concentration of 5 μM. Cells were cultured under normoxic or hypoxic conditions, with or without LPS stimulation. At the end of the experiment, adherent cells were removed from the bone slices using sodium hypochlorite. The slices were washed thoroughly with distilled water, dried, sputter-coated with gold, and examined by scanning electron microscopy. All SEM images used for quantitative comparison were acquired using consistent imaging parameters across experimental groups. Resorption lacunae were quantified using ImageJ software under identical analysis and threshold settings. Osteoclastic bone resorption was expressed as the percentage of resorbed area relative to the total analyzed bone surface area. The assay was performed using three independent biological replicates, with each replicate representing an independently performed primary BMM-derived osteoclast culture and bone-slice resorption experiment.

### 4.11 ROS and cytokine measurements

Intracellular ROS levels were measured using the DCFH-DA probe (10 μM, 30 min incubation). Intracellular ROS levels were measured using the DCFH-DA probe (10 μM, 30 min incubation) and analyzed by flow cytometry. Relative intracellular ROS levels were quantified from DCF fluorescence intensity using identical acquisition and gating settings across experimental groups. Proinflammatory cytokines (IL-6, IL-17, TNF-α) in cell supernatants and serum were determined using ELISA kits (R&D Systems, USA).

### 4.12 Ovariectomized (OVX) mouse model and treatment

All animal procedures were approved by the Institutional Animal Care and Use Committee (IACUC) of Nankai University (Protocol No. NKU-AE-2025-042). Eight-week-old female C57BL/6 mice were bilaterally ovariectomized to establish the PMOP model; sham-operated mice served as controls. After 4 weeks of model induction, mice were randomly assigned to five groups (n = 6 per group): Sham, OVX + Vehicle, OVX + PnAC4A, OVX + RLX, and OVX + RLX@PnAC4A. Treatments (equivalent RLX dose = 2 mg/kg) were administered via tail vein every 3 days for 6 weeks. The nominal RLX-equivalent dose of 2 mg/kg was selected as a literature-informed working dose within the range reported to exert skeletal effects in ovariectomized or estrogen-deficient rodent models [42,43,46].

### 4.13 In vivo bone-targeting and fluorescence imaging

To evaluate the contribution of phosphonate modification to skeletal targeting, Cy5-labeled PnAC4A was administered intravenously to OVX mice via tail-vein injection. Sham-operated mice receiving Cy5-labeled PnAC4A were included as controls. The non-phosphonated control SAC4A and free Cy5 were used as additional controls to assess the contribution of phosphonate-mediated interactions to skeletal accumulation. Whole-body fluorescence imaging was performed at the indicated time points after administration. At the designated endpoint, mice were euthanized, and the femora, tibiae, vertebrae, and major organs were harvested for ex vivo fluorescence imaging.

### 4.14 Micro-CT and histological evaluation

Femora were harvested, fixed in 4% paraformaldehyde, and scanned using micro-computed tomography (SkyScan 1276, Bruker, Belgium). Three-dimensional reconstructions and quantitative morphometric analyses were performed using CTAn software to determine bone mineral density (BMD), bone volume fraction (BV/TV), trabecular thickness (Tb.Th), trabecular number (Tb.N), trabecular separation (Tb.Sp), and cortical thickness (Ct.Th). For micro-CT analysis, a standardized region of interest was positioned 0.5 mm proximal to the distal femoral condyle. All femoral specimens were scanned using consistent acquisition parameters, and identical ROI dimensions, reconstruction procedures, segmentation thresholds, and visualization settings were applied across experimental groups. Paraffin sections (5 μm) were stained with hematoxylin–eosin (H&E), Van Gieson (VG), Safranin O/Fast Green, and TRAP. Immunohistochemistry (IHC) was conducted for OCN and CTSK to assess bone formation and resorption activity. For each histological or immunohistochemical modality, images were acquired from anatomically matched regions using the same magnification and standardized microscope settings across experimental groups. Image processing and display parameters were applied consistently within each staining modality without group-specific adjustment. Representative images of the different staining modalities were independently selected from biological replicates within each treatment group.

### 4.15 Serum biochemical and safety analysis

At the end of the 6-week treatment period, mice were euthanized, and blood samples were collected for serum biochemical analysis. Serum was isolated by centrifugation after clot formation. Hepatic function was evaluated by measuring alanine aminotransferase (ALT) and aspartate aminotransferase (AST), whereas renal function was evaluated by measuring creatinine (CREA) and urea using an automated biochemical analyzer according to the manufacturer's instructions.

The heart, liver, spleen, lung, and kidney were collected immediately after euthanasia, fixed in 4% paraformaldehyde, embedded in paraffin, and sectioned at a thickness of 5 μm. Sections were stained with hematoxylin and eosin (H&E) and examined by light microscopy for treatment-associated histopathological abnormalities, including inflammatory-cell infiltration, necrosis, hemorrhage, and disruption of tissue architecture.

### 4.16 RNA sequencing and bioinformatics analysis

Total RNA was extracted from femoral bone tissue using TRIzol reagent (Invitrogen). RNA integrity was verified (RIN > 8.0), and libraries were prepared using the NEBNext Ultra RNA Library Prep Kit for Illumina. Sequencing was performed on an Illumina NovaSeq 6000 platform with 150-bp paired-end reads. Clean reads were aligned to the *Mus musculus* reference genome (GRCm39) using HISAT2, and gene-level raw counts were generated from the aligned reads. The unnormalized integer count matrix was used as input to DESeq2 for differential expression analysis, with normalization, dispersion estimation, and negative-binomial modeling performed internally by DESeq2. Differentially expressed genes (DEGs) were defined as those with |log_2_ fold change| > 1 and a Benjamini–Hochberg-adjusted P value < 0.05. Gene Ontology (GO) and Kyoto Encyclopedia of Genes and Genomes (KEGG) enrichment analyses were performed using the clusterProfiler package in R (version 4.3.1). Gene set enrichment analysis (GSEA) was performed using the ranked gene list to identify signaling pathways coordinately enriched between the experimental groups.

### 4.17 Statistical analysis

All experiments were performed at least in triplicate. Data are presented as mean ± standard deviation (SD). Statistical analyses were conducted using GraphPad Prism 9.0. Statistical significance was assessed by one-way ANOVA followed by Tukey's multiple-comparisons test. Differences were considered statistically significant at p < 0.05.

## CRediT authorship contribution statement

**Gan Luo:** Methodology, Investigation, Formal analysis, Data curation. **Houzhi Yang:** Methodology, Investigation, Formal analysis, Data curation. **Ze-Han Wang:** Methodology, Investigation. **Jianlin Cui:** Methodology, Formal analysis. **Qiong Tang:** Methodology, Investigation. **Yonggang Fan:** Methodology. **Hong Yang:** Methodology. **Yubin Long:** Methodology. **Shanshan Li:** Supervision, Methodology, Investigation, Formal analysis. **Dong-Sheng Guo:** Supervision, Resources, Methodology, Investigation. **Tianwei Sun:** Supervision, Resources, Investigation, Funding acquisition. **Xin Jin:** Writing – review & editing, Writing – original draft, Supervision, Investigation, Funding acquisition, Formal analysis.

## Declaration of competing interest

The authors declare that they have no known competing financial interests or personal relationships that could have appeared to influence the work reported in this paper.

## Figures and Tables

**figs1:**
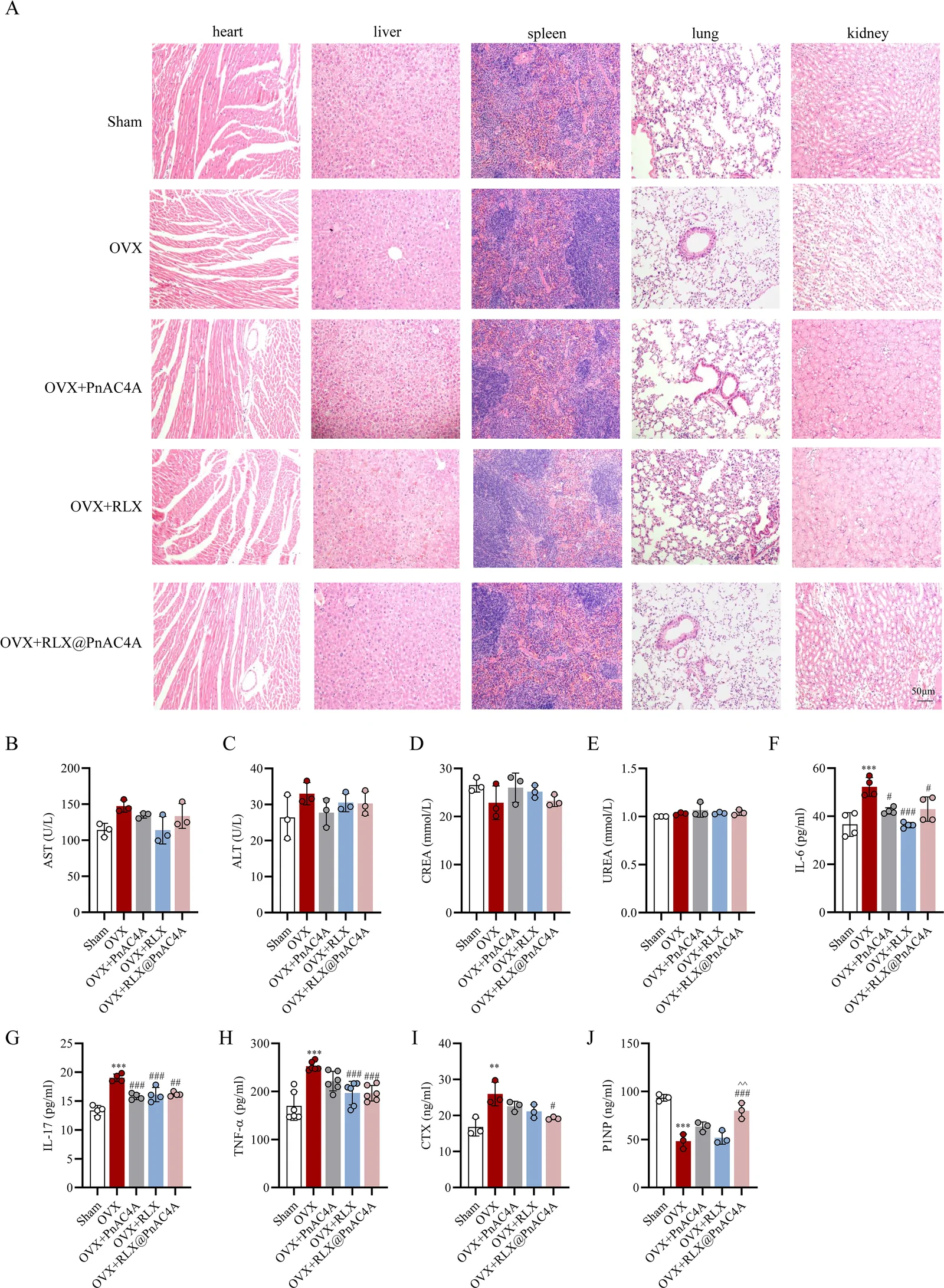


**figs2:**
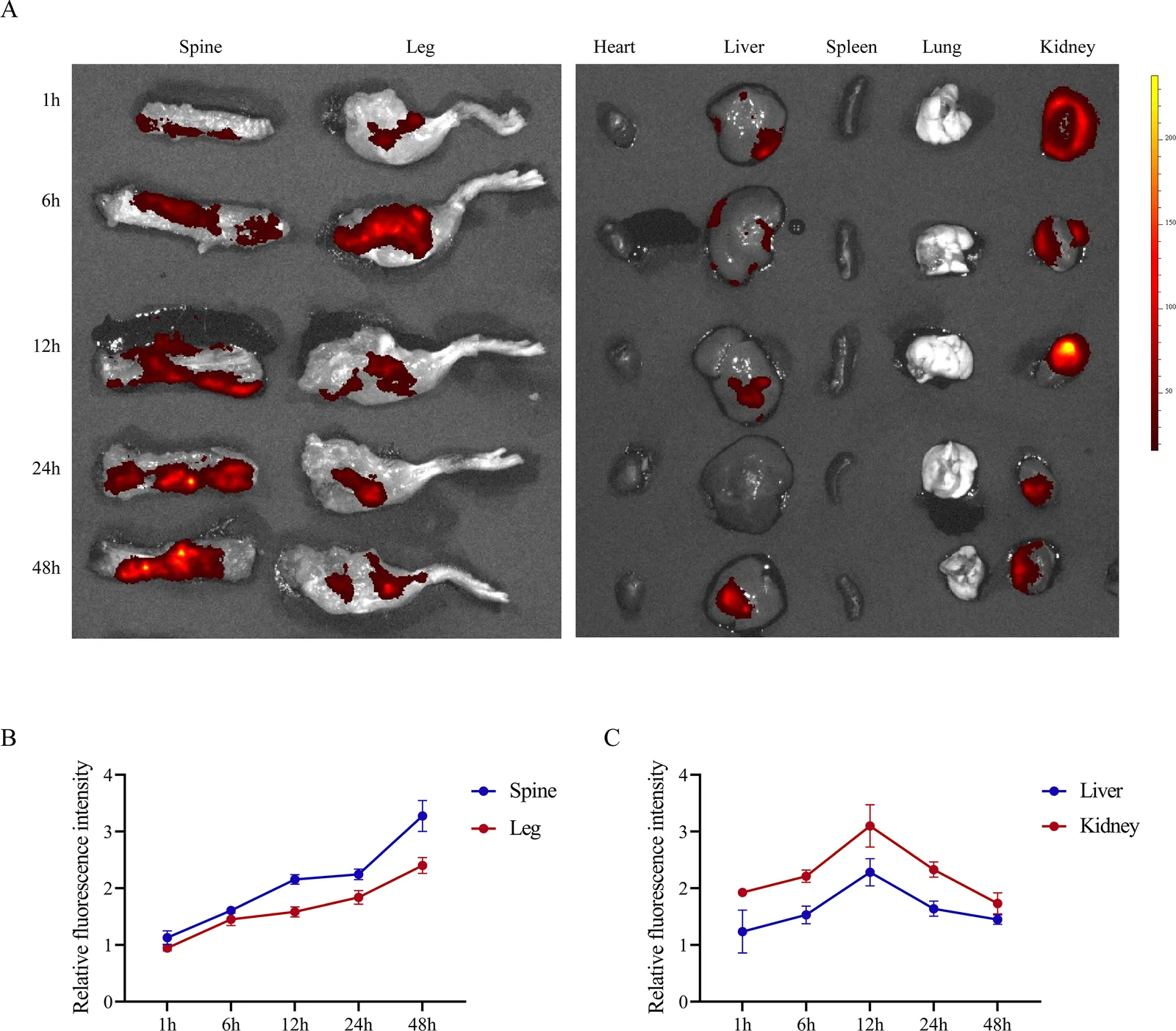


**figs3:**
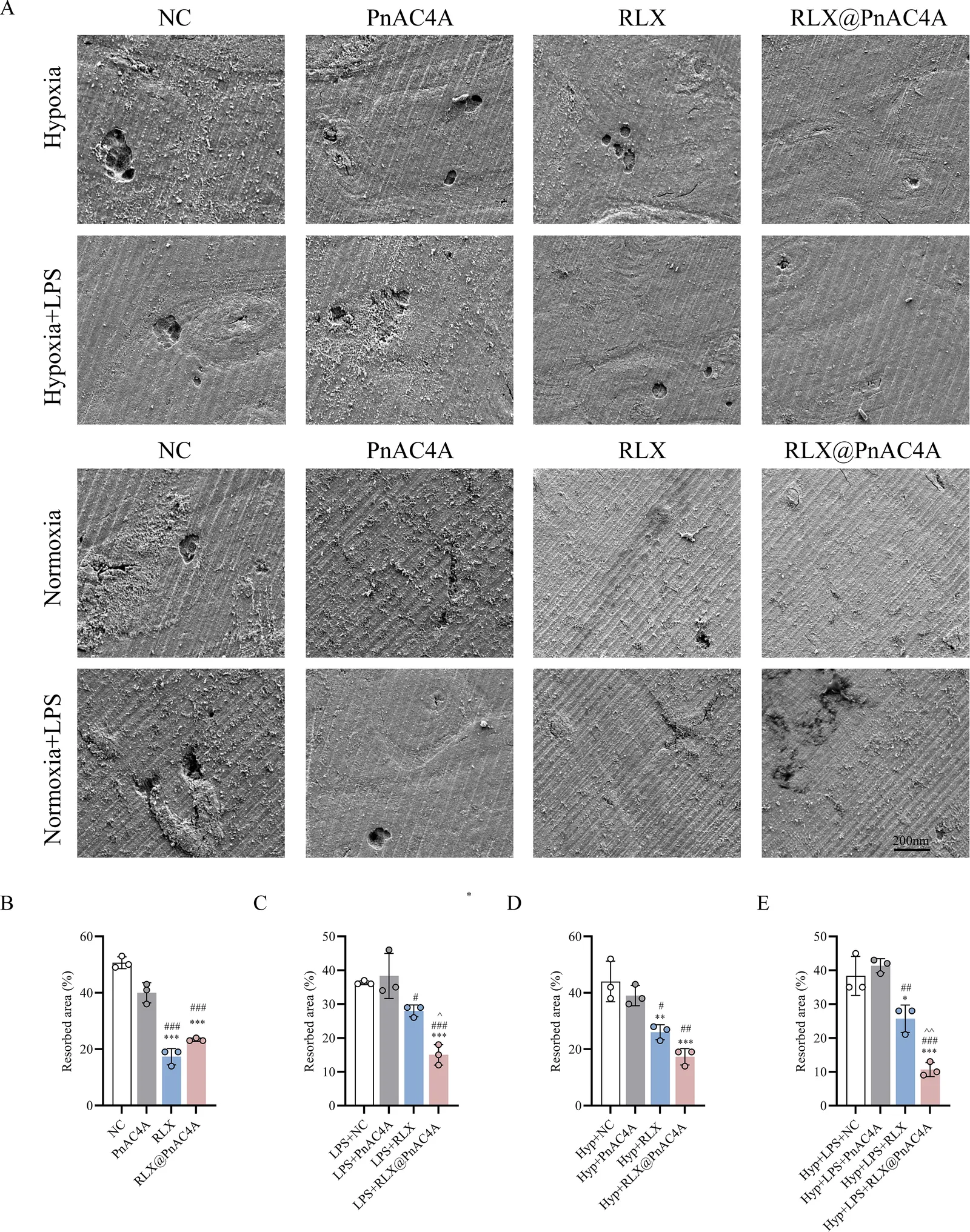


**figs4:**
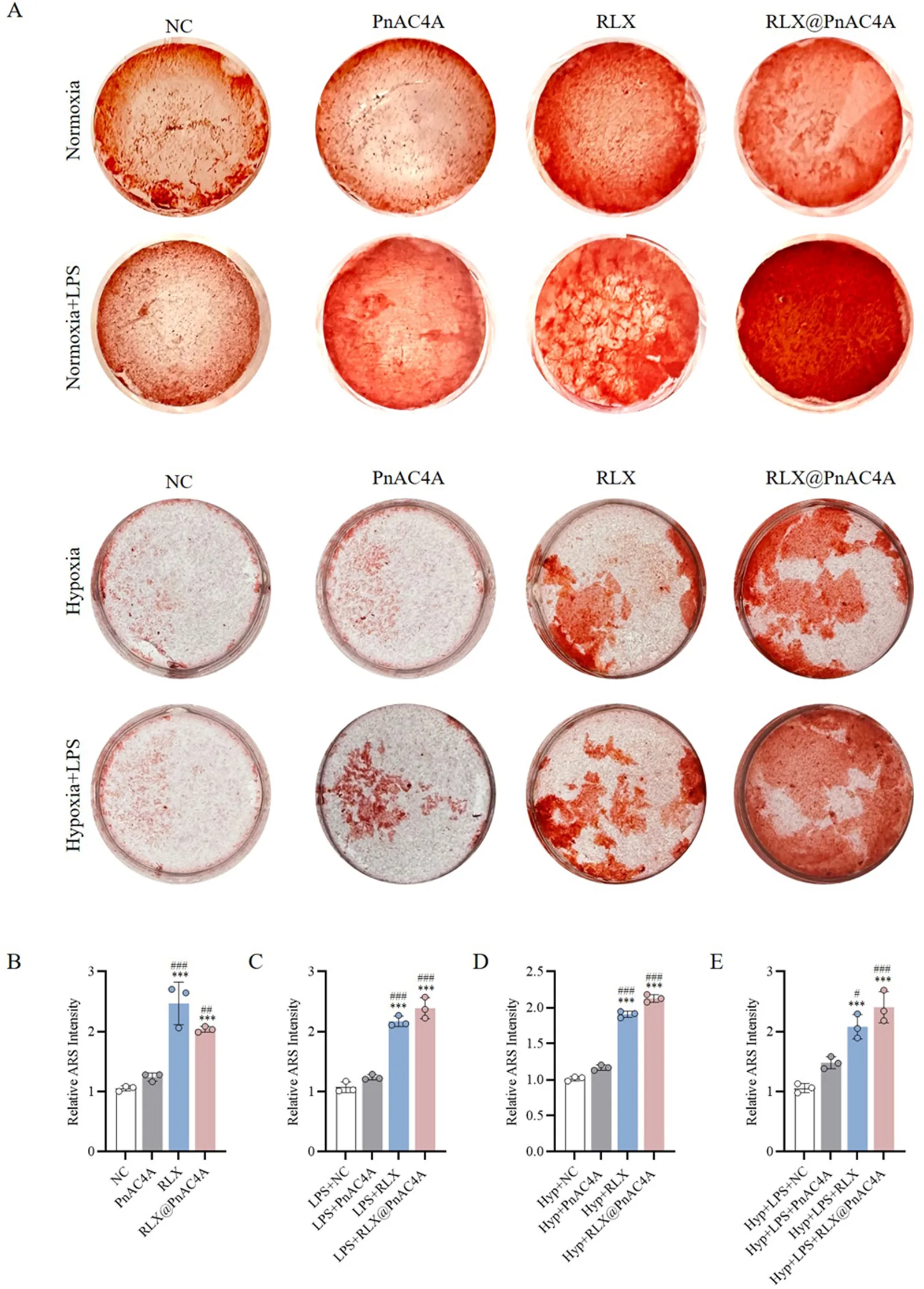


**figs5:**
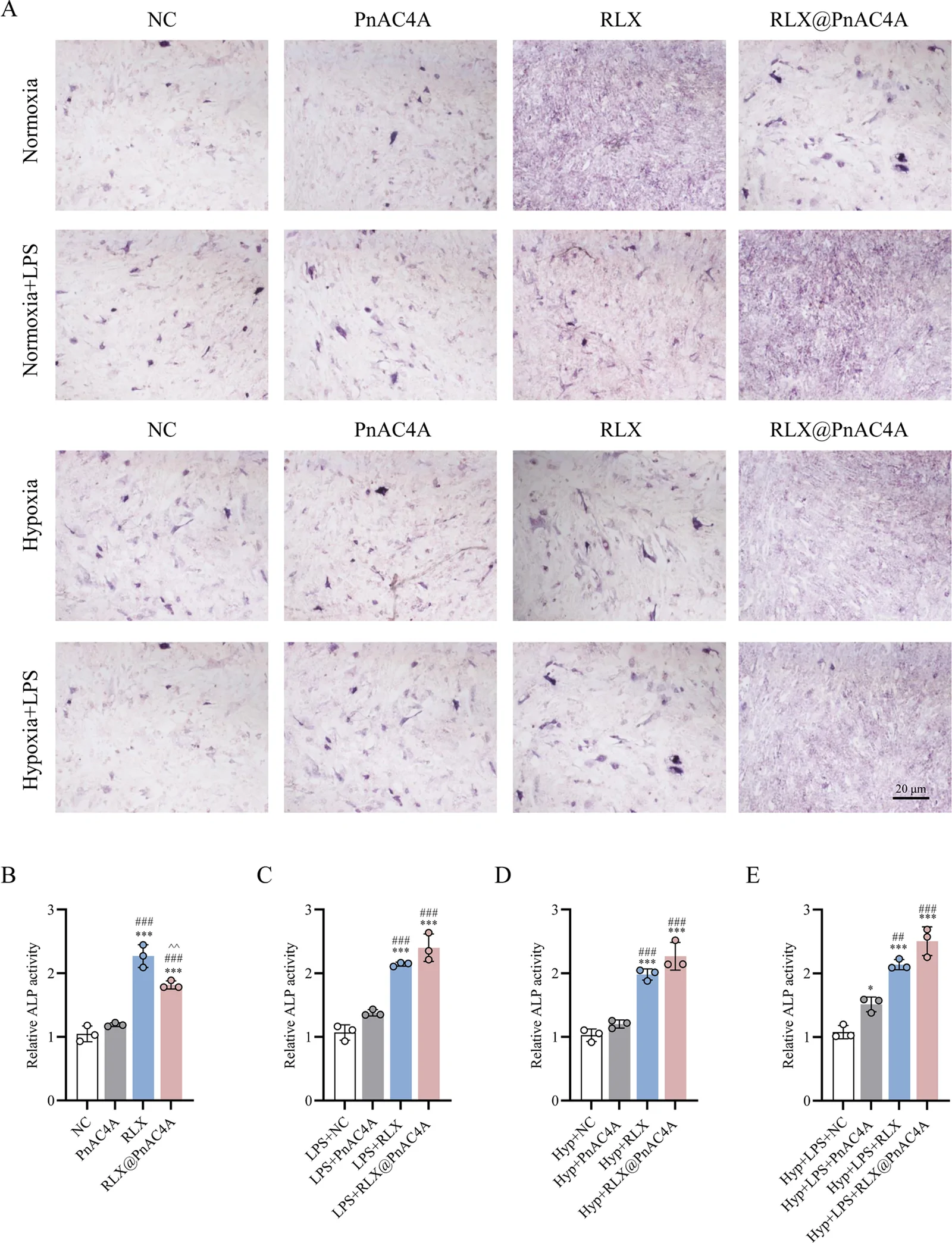


**figs6:**
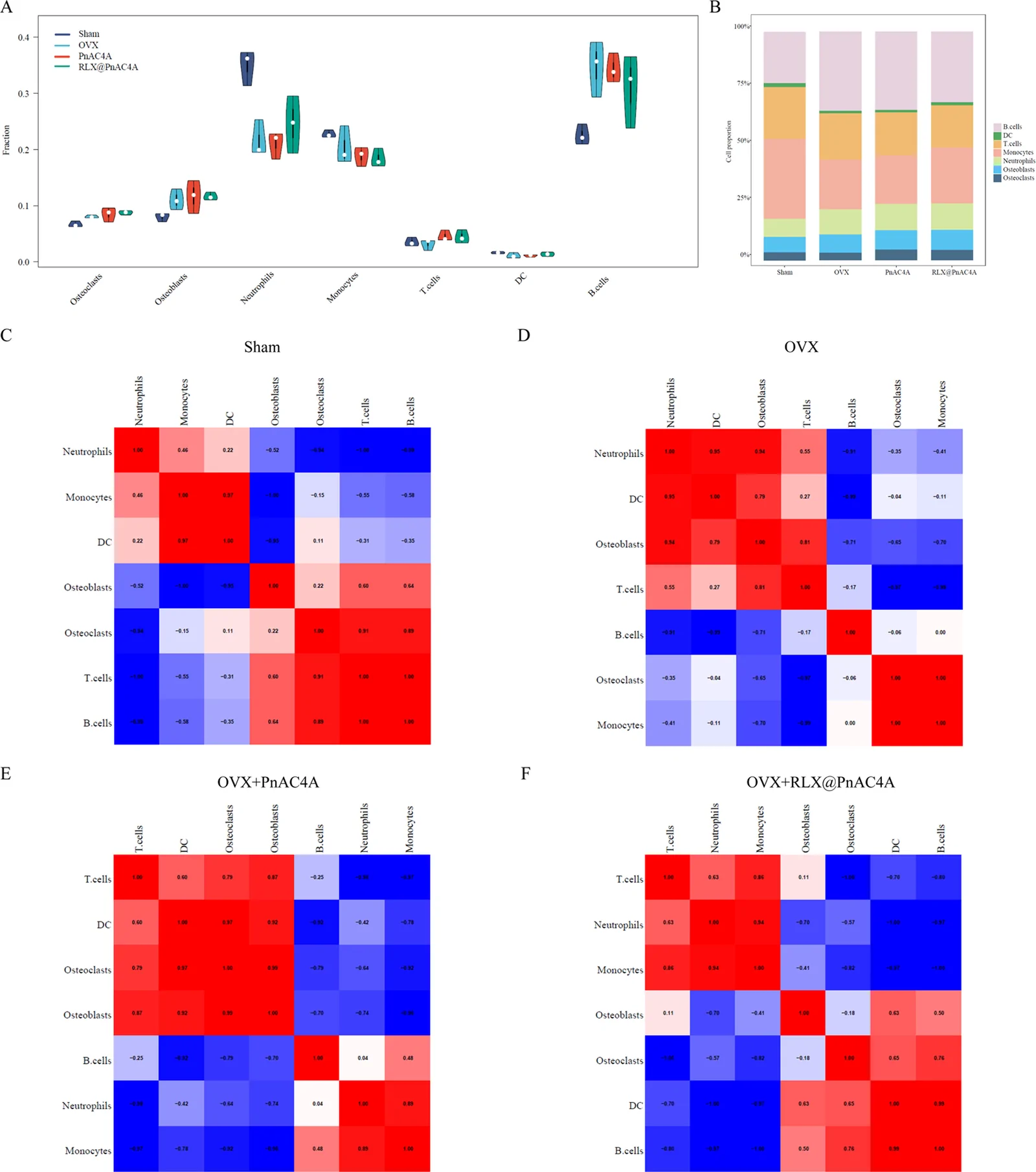


**figs7:**
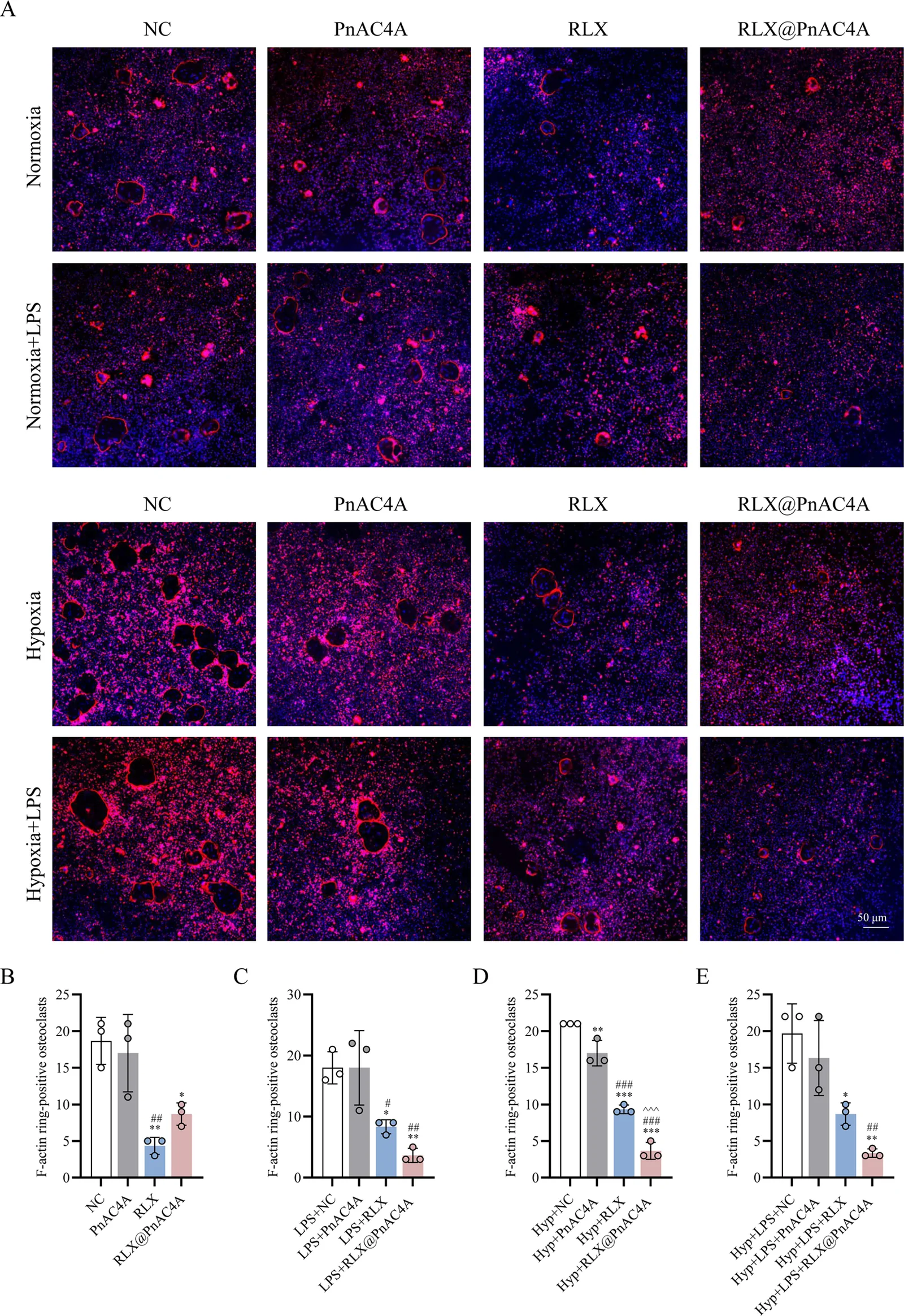


**figs8:**
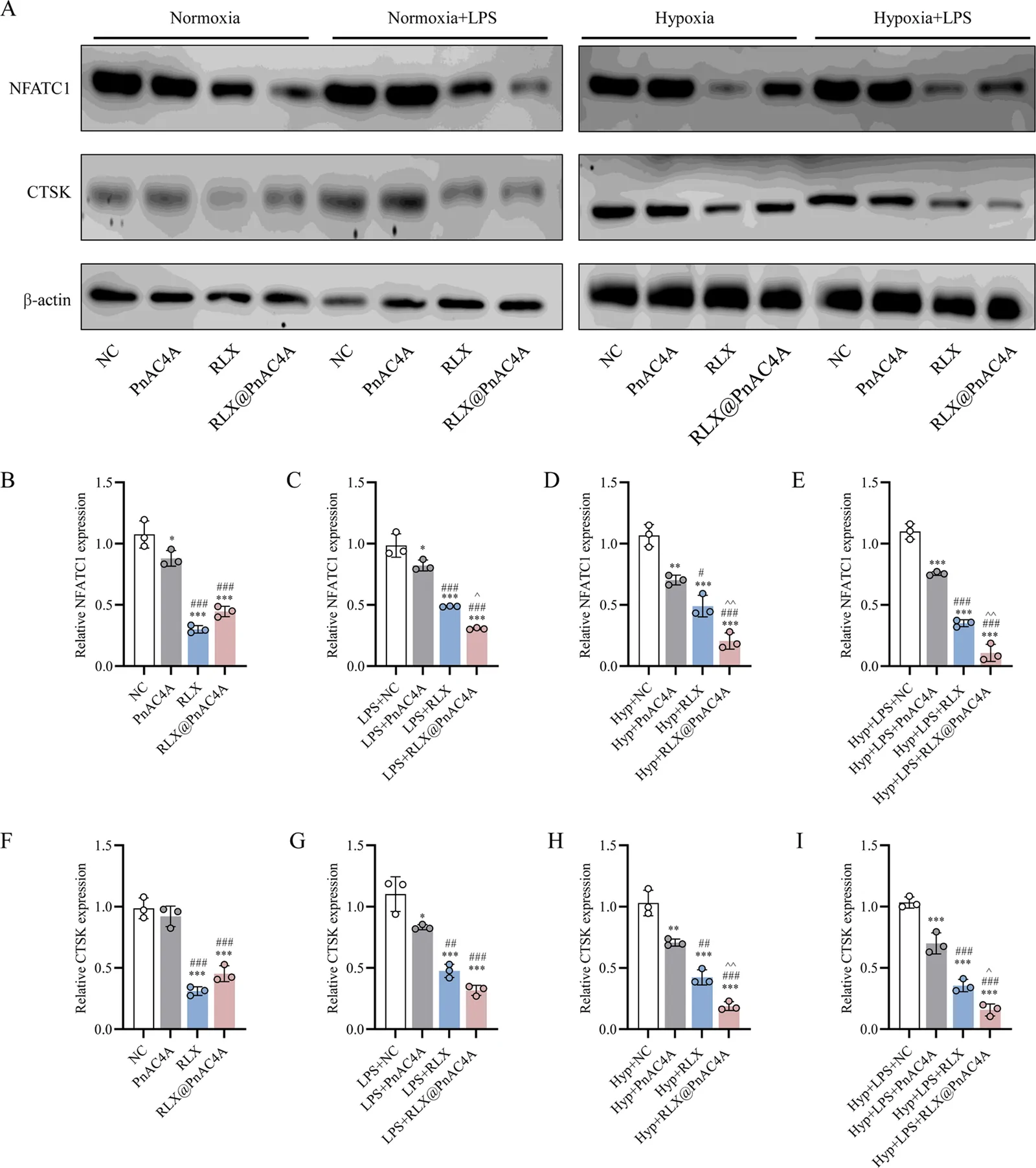


## Data Availability

Data will be made available on request.

## References

[1] Liang H. (2025). Global epidemiology and burden of osteoporosis among postmenopausal women: insights from the Global burden of disease study 2021. NPJ Aging.

[2] Salimi M. (2024). Association of neutrophil to lymphocyte ratio with bone mineral density in post-menopausal women: a systematic review and meta-analysis. BMC Womens Health.

[3] Chen M. (2025). The interplay of estrogen, gut microbiome, and bone immunity in osteoporosis. Cell Commun. Signal..

[4] Hansdah K., Lui J.C. (2024). Emerging insights into the endocrine regulation of bone homeostasis by gut microbiome. J. Endocr. Soc..

[5] Chen L. (2024). Analysis and validation of biomarkers of immune cell-related genes in postmenopausal osteoporosis: an observational study. Medicine (Baltim.).

[6] Luo J. (2025). Oxidative stress and inflammation: roles in osteoporosis. Front. Immunol..

[7] Yin X. (2019). Autophagy in bone homeostasis and the onset of osteoporosis. Bone Res..

[8] Qaseem A. (2023). Pharmacologic treatment of primary osteoporosis or low bone mass to prevent fractures in adults: a living clinical guideline from the American college of physicians. Ann. Intern. Med..

[9] Kumar S. (2025). Denosumab discontinuation in the clinic: implications of rebound bone turnover and emerging strategies to prevent bone loss and fractures. J. Bone Miner. Res..

[10] Sendhil S.R. (2025). Follow-on osteoporosis therapy after denosumab discontinuation among medicare beneficiaries. Osteoporos. Int..

[11] Lee C.C. (2023). The real-world adherence of the first-line anti-osteoporosis medications in Taiwan: visualize the gap between reality and expectations. J. Formos. Med. Assoc..

[12] Smith A. (2025). Utilization and expenditure on medicines for the management of osteoporosis in Ireland: a repeated cross-sectional study. Br. J. Clin. Pharmacol..

[13] Handel M.N. (2023). Fracture risk reduction and safety by osteoporosis treatment compared with placebo or active comparator in postmenopausal women: systematic review, network meta-analysis, and meta-regression analysis of randomised clinical trials. BMJ.

[14] Ettinger B. (1999). Reduction of vertebral fracture risk in postmenopausal women with osteoporosis treated with raloxifene: results from a 3-year randomized clinical trial. Multiple Outcomes of Raloxifene Evaluation (MORE) investigators. JAMA.

[15] A.L J.K. (2025). Raloxifene-loaded lipid nanovesicles: a journey to select the optimal nanocarrier formulation through characterization and cytotoxic analysis. Biomedicines.

[16] Daponte V., Henke K., Drissi H. (2024). Current perspectives on the multiple roles of osteoclasts: mechanisms of osteoclast-osteoblast communication and potential clinical implications. eLife.

[17] Wang W. (2026). A pseudo-mytilus edulis foot protein-based hydrogel adhesive with osteo-vascular-immune coupling effects for osteoporotic bone-implant integration. Adv. Mater..

[18] Zhou W. (2024). Mussel-derived and bioclickable peptide mimic for enhanced interfacial osseointegration via synergistic immunomodulation and vascularized bone regeneration. Adv. Sci. (Weinh.).

[19] Ma X. (2025). Recent advances in supramolecular systems for precision medicine: structural design, functional integration, and clinical translation challenges. Pharmaceutics.

[20] Ji J. (2023). Asymmetric photoreactions in supramolecular assemblies. Acc. Chem. Res..

[21] Hu W. (2026). Stimuli-responsive supramolecular biomaterials for cancer theranostics. Adv. Sci. (Weinh.).

[22] Pan Y.C., Hu X.Y., Guo D.S. (2021). Biomedical applications of calixarenes: state of the art and perspectives. Angew Chem. Int. Ed. Engl..

[23] Guo D.S., Liu Y. (2014). Supramolecular chemistry of p-sulfonatocalix[n]arenes and its biological applications. Acc. Chem. Res..

[24] Yu G., Chen X. (2019). Host-guest chemistry in supramolecular theranostics. Theranostics.

[25] Luan X. (2023). Silane-functionalized metal-organic frameworks for stimuli-responsive drug delivery systems: a new universal strategy. ACS Appl. Mater. Interfaces.

[26] Shetty D. (2019). Self-assembly of stimuli-responsive imine-linked calix[4]arene nanocapsules for targeted camptothecin delivery. Chem. Commun..

[27] Ranamalla S.R. (2022). An overview of the supramolecular systems for gene and drug delivery in tissue regeneration. Pharmaceutics.

[28] Zhang T.X. (2022). A hypoxia-responsive supramolecular formulation for imaging-guided photothermal therapy. Theranostics.

[29] Russell R.G. (2008). Mechanisms of action of bisphosphonates: similarities and differences and their potential influence on clinical efficacy. Osteoporos. Int..

[30] Drake M.T., Clarke B.L., Khosla S. (2008). Bisphosphonates: mechanism of action and role in clinical practice. Mayo Clin. Proc..

[31] Hsu S.H., Chen L.R., Chen K.H. (2024). Primary osteoporosis induced by androgen and estrogen deficiency: the molecular and cellular perspective on pathophysiological mechanisms and treatments. Int. J. Mol. Sci..

[32] Li M. (2025). Pro-inflammatory immune microenvironment and Thrombospondin-1-positive monocytes as drivers of osteoclastogenesis in postmenopausal osteoporosis. J. Bone Miner. Res..

[33] Terhaar H. (2025). The crosstalk between B cells and the skeletal system during development, aging, and in pathological conditions. Curr. Osteoporos. Rep..

[34] Meng X. (2022). Estrogen-mediated downregulation of HIF-1alpha signaling in B lymphocytes influences postmenopausal bone loss. Bone Res..

[35] Kimball J.S., Johnson J.P., Carlson D.A. (2021). Oxidative stress and osteoporosis. J. Bone Joint Surg. Am..

[36] Wu Q. (2024). Tetrahydroberberine prevents ovariectomy-induced bone loss by inhibiting RANKL-induced osteoclastogenesis and promoting osteoclast apoptosis. J. Agric. Food Chem..

[37] Yu C. (2025). Idebenone attenuates RANKL-induced osteoclastogenesis and improves bone mass in ovariectomized mice. Free Radic. Biol. Med..

[38] Zhang Z. (2025). Single-cell transcriptomic analysis reveals AP-1 downregulation remodels bone marrow environment and contributes to osteopenia in ovariectomized mice. J. Orthop. Transl..

[39] Li S. (2025). Cell communication and relevant signaling pathways in osteogenesis-angiogenesis coupling. Bone Res..

[40] Wu Q. (2026). Designed bone-targeting ROS-responsive nanoplatform for precision glycolysis inhibition in postmenopausal osteoporosis. Bioact. Mater..

[41] Guo Z. (2023). Investigation of the treatment potential of Raloxifene-loaded polymeric nanoparticles in osteoporosis: in-vitro and in-vivo analyses. Heliyon.

[42] Black L.J. (1994). Raloxifene (LY139481 HCI) prevents bone loss and reduces serum cholesterol without causing uterine hypertrophy in ovariectomized rats. J. Clin. Investig..

[43] Cheng Z. (2009). Prolonged treatments with antiresorptive agents and PTH have different effects on bone strength and the degree of mineralization in old estrogen-deficient osteoporotic rats. J. Bone Miner. Res..

[44] Hioki T. (2022). Selective estrogen receptor modulators, acting as agonists of estrogen receptor alpha in osteoblasts, reduce the TGF-beta-induced synthesis of macrophage colony-stimulating factor via inhibition of JNK signaling pathway. Biomed. Res..

[45] Morita M. (2016). Selective estrogen receptor modulators suppress Hif1alpha protein accumulation in mouse osteoclasts. PLoS One.

[46] Zhou L.P. (2018). Bone protective effects of Danggui Buxue Tang alone and in combination with tamoxifen or raloxifene in vivo and in vitro. Front. Pharmacol..

[47] Wang Ze-Han, C Y.-Q., Li Wen-Bo, Chen Meng-Meng, Zhang Shu-Xin, Pan Yu-Chen, Guo Dong-Sheng (2025). A study of chiral biomedical effects based on macrocyclic carriers. CCS Chem..

