# Supplementary material for: A hypoxia-responsive calixarene supramolecular system enables redox-regulated matrix remodeling for postmenopausal osteoporosis
Source: Mater Today Bio. 2026 Sep 23;41:103685. doi: 10.1016/j.mtbio.2026.103685 (PMC13659505; doi:10.1016/j.mtbio.2026.103685)
Supplement: Supplementary file 1 [file mmc1.docx]

**Supplementary Figure S1. Systemic tolerability of repeated RLX@PnAC4A administration in OVX mice.** (A) Representative hematoxylin and eosin (H&E)-stained sections of the heart, liver, spleen, lung, and kidney collected from the Sham, OVX, OVX+PnAC4A, OVX+RLX, and OVX+RLX@PnAC4A groups at the end of the 6-week treatment period. No overt treatment-associated histopathological abnormalities were observed in the major organs. For each organ, representative H&E images were acquired at the same magnification using consistent imaging and display parameters across experimental groups. (B–E) Serum biochemical indices of hepatic and renal function, including (B) aspartate aminotransferase (AST), (C) alanine aminotransferase (ALT), (D) creatinine (CREA), and (E) urea. (F–H) Serum concentrations of the pro-inflammatory cytokines IL-6 (F), IL-17 (G), and TNF-α (H). (I–J) Serum levels of the bone-resorption marker CTX-I (I) and the bone-formation marker P1NP (J). Data are presented as mean ± SD from n = 3 mice per group. *P < 0.05, **P < 0.01, ***P < 0.001 vs. Sham; #P < 0.05, ##P < 0.01, ###P < 0.001 vs. OVX; ^P < 0.05, ^^P < 0.01, ^^^P < 0.001 vs. OVX+RLX.

**Supplementary Figure S2. Time-dependent ex vivo biodistribution of Cy5-labeled PnAC4A in ovariectomized mice.** (A) Representative ex vivo fluorescence images of skeletal tissues and major organs collected at 1, 6, 12, 24, and 48 h after tail-vein administration of Cy5-labeled PnAC4A to ovariectomized mice. The analyzed tissues included the spine, hindlimb, heart, liver, spleen, lung, and kidney. All tissues were imaged using identical acquisition and display settings. (B) Quantification of the relative fluorescence intensity in the spine and hindlimb at the indicated time points, showing progressive skeletal accumulation and sustained retention of Cy5-labeled PnAC4A. (C) Quantification of the relative fluorescence intensity in the liver and kidney. Fluorescence signals in these clearance-associated organs increased during the early distribution phase, peaked at approximately 12 h, and subsequently declined. Data are presented as mean ± SD from n = 6 mice/time point.

**Supplementary Figure S3. RLX@PnAC4A inhibits osteoclast-mediated bone resorption under hypoxic and inflammatory conditions.** (A) Representative scanning electron microscopy images of resorption lacunae formed by osteoclasts on bovine cortical bone slices under normoxia, normoxia plus LPS, hypoxia, and hypoxia plus LPS following treatment with NC, PnAC4A, free RLX, or RLX@PnAC4A. (B–E) Quantification of the resorbed surface area under normoxia (B), normoxia plus LPS (C), hypoxia (D), and hypoxia plus LPS (E). Resorption was expressed as the percentage of resorbed area relative to the total analyzed bone surface. Representative SEM images were acquired using consistent imaging parameters across experimental groups. Data are presented as mean ± SD from n = 3 independent biological replicates. Within each environmental condition, statistical significance among the NC, PnAC4A, RLX, and RLX@PnAC4A groups was assessed by one-way ANOVA followed by Tukey’s multiple-comparisons test. *P < 0.05, **P < 0.01, ***P < 0.001 vs. NC; #P < 0.05, ##P < 0.01, ###P < 0.001 vs. PnAC4A; ^P < 0.05, ^^P < 0.01, ^^^P < 0.001 vs. RLX.

**Supplementary Figure S4. RLX@PnAC4A promotes matrix mineralization in primary mouse BMSCs under hypoxic and inflammatory stress.** (A) Representative whole-well images of Alizarin Red S staining in primary mouse BMSCs cultured under normoxia, normoxia plus LPS, hypoxia, or hypoxia plus LPS following treatment with vehicle control (NC), PnAC4A, free RLX, or RLX@PnAC4A. (B–E) Quantification of Alizarin Red S staining under normoxia (B), normoxia plus LPS (C), hypoxia (D), and hypoxia plus LPS (E). Data are presented as mean ± SD from n = 3 independent experiments. *P < 0.05, **P < 0.01, ***P < 0.001 vs. NC; #P < 0.05, ##P < 0.01, ###P < 0.001 vs. PnAC4A; ^P < 0.05, ^^P < 0.01, ^^^P < 0.001 vs. RLX.

**Supplementary Figure S5. RLX@PnAC4A enhances early osteogenic differentiation of primary mouse BMSCs under hypoxic and inflammatory conditions.** (A) Representative images of alkaline phosphatase staining in primary mouse BMSCs cultured under normoxia, normoxia plus LPS, hypoxia, or hypoxia plus LPS following treatment with NC, PnAC4A, free RLX, or RLX@PnAC4A. (B–E) Quantification of ALP staining under normoxia (B), normoxia plus LPS (C), hypoxia (D), and hypoxia plus LPS (E). Data are presented as mean ± SD from n = 3 independent experiments. *P < 0.05, **P < 0.01, ***P < 0.001 vs. NC; #P < 0.05, ##P < 0.01, ###P < 0.001 vs. PnAC4A; ^P < 0.05, ^^P < 0.01, ^^^P < 0.001 vs. RLX.

**Supplementary Figure S6. CIBERSORTx-based deconvolution analysis reveals cellular composition remodeling after RLX@PnAC4A treatment.** (A) Estimated fractions of major bone-associated cell populations inferred by CIBERSORTx across Sham, OVX, PnAC4A, and RLX@PnAC4A groups. Seven representative cell populations were evaluated, including osteoblasts, osteoclasts, neutrophils, monocytes, dendritic cells, T cells, and B cells. (B) Relative composition of inferred cellular populations across experimental groups. (C–F) Correlation heatmaps showing relationships among inferred cell fractions in Sham (C), OVX (D), OVX+PnAC4A (E), and OVX+RLX@PnAC4A (F) groups.

**Supplementary Figure S7. RLX@PnAC4A disrupts F-actin ring formation and cytoskeletal polarization in osteoclasts.** (A) Representative fluorescence images of F-actin organization in RANKL-induced primary BMM-derived osteoclasts treated with vehicle, PnAC4A, free RLX, or RLX@PnAC4A under normoxia, normoxia plus LPS, hypoxia, and hypoxia plus LPS conditions. F-actin was visualized using fluorescent phalloidin staining (magenta/red), and nuclei were counterstained with DAPI (blue).

(B–E) Quantification of F-actin ring-positive multinucleated osteoclasts under normoxia (B), normoxia plus LPS (C), hypoxia (D), and hypoxia plus LPS (E). Multinucleated cells containing at least three nuclei and displaying a continuous or near-continuous peripheral F-actin ring were counted as F-actin ring-positive osteoclasts. Data are presented as mean ± SD from n = 3 independent experiments.

**Supplementary Figure S8. RLX@PnAC4A suppresses osteoclastogenic protein expression under hypoxic and inflammatory conditions.** (A) Representative Western blot images of NFATc1 and cathepsin K (CTSK) in RANKL-induced primary bone marrow–derived macrophage (BMM)-derived osteoclasts treated with vehicle control (NC), PnAC4A, free raloxifene (RLX), or RLX@PnAC4A under normoxia, normoxia plus LPS, hypoxia, or hypoxia plus LPS. β-Actin was used as the loading control. (B–E) Densitometric quantification of NFATc1 protein abundance under normoxia (B), normoxia plus LPS (C), hypoxia (D), and hypoxia plus LPS (E). (F–I) Densitometric quantification of CTSK protein abundance under normoxia (F), normoxia plus LPS (G), hypoxia (H), and hypoxia plus LPS (I). Protein abundance was normalized to β-actin and expressed relative to the corresponding NC group. Data are presented as mean ± SD from n = 3 independent experiments. P < 0.05, P < 0.01, and P < 0.001 vs. NC; #P < 0.05, ##P < 0.01, and ###P < 0.001 vs. PnAC4A; ^P < 0.05, ^^P < 0.01, and ^^^P < 0.001 vs. RLX.
